# Prebiotics attenuate depressive-like behavior, neuroinflammation and synaptic plasticity in Parkinson’s disease by modulating butyrate-producing gut bacteria

**DOI:** 10.1007/s10787-026-02152-2

**Published:** 2026-03-13

**Authors:** Ingrid Prata de Mendonça, Rodrigo Soares da Silva, Igor Henrique Rodrigues de Paiva, Belmira Lara da S. A. Costa, Karla Patrícia de Sousa Barbosa Teixeira, José Roberto Botelho de Souza, Christina Alves Peixoto

**Affiliations:** 1 Laboratory of Ultrastructure, Aggeu Magalhães Institute (IAM), Recife, PE Brazil; 2 Postgraduate Program in Biological Sciences/Center of Biosciences, Federal University of Pernambuco (UFPE), Recife, PE Brazil; 3 Laboratory of Neurophysiology - Department of Physiology and Pharmacology, Federal University of Pernambuco, Recife, PE Brazil; 4 Academic Center of Vitoria (CAV), Federal University of Pernambuco, Recife, PE Brazil; 5 Department of Zoology, Federal University of Pernambuco, Recife, PE Brazil; 6 Institute of Science and Technology on Neuroimmunomodulation (INCT-NIM), Rio de Janeiro, Brazil

**Keywords:** Parkinson’s disease, Gut-brain axis, Butyrate, Depression, Neuroinflammation

## Abstract

Parkinson’s disease (PD) remains a challenging disease for treatment, which is usually polypharmacological. In addition to motor symptoms, non-motor symptoms such as depression are present in approximately 40% of patients, contributing to the loss of quality of life. In the last two decades, a growing body of evidence has emerged regarding the involvement of the microbiota-gut-brain axis in both PD and depression. Fructooligosaccharides (FOS) and galactooligosaccharides (GOS) are prebiotic fibers that can be fermented by the gut microbiota, which produce metabolites called short-chain fatty acids (SCFAs), whose effects can contribute to improvement in neurodegenerative and psychiatric conditions. This study analyzed the effects of FOS and GOS administration in a rotenone-induced PD model and demonstrated a relief of motor symptoms and depressive-like behavior, followed by an increase of brain serotonin and its respective receptor (SERT). FOS and GOS treatment also led to an increase in SCFAs-producing gut bacteria with significantly higher levels of serum and brain butyrate. Furthermore, in the intestine, prebiotics reduced the accumulation of α-synuclein, decreased inflammation, and improved the expression of zonula occludens and occludin. FOS and GOS also attenuated the loss of dopaminergic neurons and reduced neuroinflammation by decreasing α-synuclein, IBA-1, GFAP, iNOS, p-NFkB, and IL1-β levels in the substantia nigra and prefrontal cortex. In addition, these prebiotics improved neuroplasticity by promoting the expression of butyrate receptors (GPR43 and GPR109), BDNF, p-CREB, and synaptic protein PSD-95. In conclusion, FOS and GOS administration attenuatted depressive-like behavior, neuroinflammation, and synaptic plasticity in Parkinson’s disease by modulating butyrate-producing gut bacteria.

## Introduction

Parkinson’s disease (PD) is a chronic pathology of the central nervous system (CNS) that affects approximately 1% of the world’s population over 65 years of age and is considered the second most prevalent neurodegenerative disease in the world (Ascherio and Schwarzschild 2016). PD is also known as paralysis agitans and results mainly from the progressive destruction of neurons in the substantia nigra pars compacta (SNpc), which sends dopamine (DA)-secreting nerve fibers to the basal nuclei of the striatum: caudate and putamen (Tysnes and Storstein 2017). The etiology of PD has been the subject of intense investigation and has long remained unclear. Currently, studies suggest that, in addition to genetic factors and exposure to environmental toxins, conditions such as diabetes mellitus and obesity may increase the risk of developing PD due to insulin resistance in the brain, which may compromise the dopamine signaling pathway (Brudek 2019; Fiory et al. 2019).

Clinically, PD is characterized by (1) motor symptoms such as stiffness of a large part of the body’s muscles, intense difficulty in initiating movements known as bradykinesia, postural instability, leading to imbalance and frequent falls, and tremors in the extremities, even when the person is at rest; and (2) non-motor symptoms such as gastrointestinal dysfunction, depression, sleep disorders and cognitive deficit (Guyton and Hall. 2011).

Depression affects approximately 30–40% of patients with PD and is the main non-motor symptom involving the CNS. Among patients who are treated for depression, half remain depressed, suggesting treatment ineffectiveness (Frisina et al. 2009; Bhattacharjee et al. 2018). Depression is associated with a central nervous system (CNS) immunoinflammatory disease characterized by increased expression of pro-inflammatory cytokines, oxidative and nitrosative stress (Moylan et al. 2013, 2014).

In addition, studies suggest that the peripheral nervous system (PNS) and enteric nervous system (ENS) may also be involved in the neurodegenerative process underlying PD. Gastrointestinal (GI) dysfunction is a common non-motor symptoms affecting approximately 80% of patients, and it can occur at any stage of PD, including years before diagnosis (Johnson et al. 2018; Zhang et al. 2023). According to the Braak hypothesis, the inflammatory microenvironment and accumulation of toxic α-synuclein, may occur in the gut decades before the onset of motor symptoms and subsequently reach the brain through gut-brain axis communication (Braak and Del Tredici 2017; Van Kessel and El Aidy 2019).

The bacteria belonging to the gut microbiota perform essential functions for maintaining the individual’s health; however, changes in the composition and function of the gut microbiota, characterized by a reduction in beneficial bacteria and an increase in potentially harmful microorganisms (gut dysbiosis), are implicated in several inflammatory diseases, such as ulcerative colitis (Aas et al. 2003), diabetes (Karlsson et al. 2013), asthma (Abrahamsson et al. 2014), and in neuropsychiatric and neurodegenerative diseases including Parkinson’s disease, Alzheimer’s disease, multiple sclerosis, autism, and depression (Fond et al. 2015; Hasegawa et al. 2015; Quigley 2017; Hirayama and Ohno 2021).

Gut dysbiosis can induce systemic inflammation by releasing high levels of endotoxin (LPS) that induce the secretion of pro-inflammatory cytokines, which can reach the CNS glia (Cario and Podolsky 2005). This altered gut microbiota may be linked to gastrointestinal disturbances in PD patients, along with “leaky gut” and intestinal inflammation, which occur long before motor disturbances (Hill-Burns et al. 2017; Lin et al. 2018; Aho et al. 2019). Furthermore, gut dysbiosis contributes to α-synuclein aggregation, loss of tyrosine hydroxylase-positive neurons in the substantia nigra, and motor symptoms in a rotenone-induced murine model of Parkinson’s disease (Fang et al. 2024).

Recently, some therapeutic interventions to modulate intestinal microbiota have been proposed, such as probiotics, prebiotics, synbiotics, and even fecal microbiota transplantation. Each approach has advantages and disadvantages, but all have shown significant potential for improving PD symptoms in patients and animal models (Gazerani 2019; Peterson 2020; Zhao et al. b).

Prebiotics are carbohydrates not digestible by human cells, which are fermented by several groups of bacteria in the intestinal microbiota. The fermentation of these carbohydrates produces metabolites called short-chain fatty acids (SCFAs). In the human intestine, acetate, propionate, and butyrate represent approximately 95% of all SCFAs (Ríos-Covián et al. 2016). SCFAs play important physiological roles, such as maintaining the integrity of the intestinal epithelial barrier, immune regulation, and providing energy for colonocytes (Tan et al. 2014).

The prebiotics fructooligosaccharides (FOS) and galactooligosaccharides (GOS) are diety fibers that promote the growth of beneficial bacteria (Burokas et al. 2017; Paiva et al. 2020; de Paiva et al. 2023). Furthermore, data obtained in several studies suggest that prebiotics promote antidepressant and anxiolytic effects by modulating the intestinal microbiota (Paiva et al. 2020). We hypothesized that FOS and GOS have beneficial effects on PD-associated depressive-like behavior in C57BL6 mice by modulating SCFA production. To investigate this further, we conducted a comprehensive study using rotenone-induced C57BL6 mice, a well-established model for Parkinson’s disease, to analyze the impact of FOS and GOS treatment on depressive-like behavior and explore the underlying molecular mechanisms.

## Methodology

### Animals

Male mice of the isogenic C57BL/6 line were obtained from the Aggeu Magalhães Institute (IAM-FIOCRUZ) vivarium at the seventh week of age. The animals were acclimated to a temperature of 18–22 °C in microisolators containing 4 animals each. The experimental design of this study is in accordance with the Ethical Principles in Animal Experimentation and was approved by the Ethics Committee on the Use of Animals of the IAM by protocol number 169/2021.

### Experimental design

Forty animals were divided into four experimental groups (*n* = 10/group):

1. CONTROL—animals subjected to the vehicle solution subcutaneously
2. PD—animals induced with rotenone subcutaneously
3. PD + PREBIOTICS—animals induced with rotenone subcutaneously and treated orally with FOS and GOS
4. PD + FLUOXETINE—animals induced with rotenone subcutaneously and and treated orally with fluoxetine

Rotenone was diluted in a vehicle solution containing 98% sunflower oil + 2% DMSO and administered subcutaneously at a dose of 2.5 mg/kg/day. FOS and GOS were diluted in sterile water and orally administered (gavage) at 3 g/kg/day and 4 g/kg/day, respectively. Rotenone, FOS and GOS were administered simultaneously for 20 consecutive days. Fluoxetine was used as a pattern antidepressive drug; it was diluted in sterile water and administered at a dose of 10 mg/kg/day simultaneously with rotenone for 20 consecutive days.

All the animals were subjected to the rotarod test, open field test (motor dysfunction evaluation), tail suspension test, and sucrose preference test (evaluation of depressive-like behavior), and no animals were excluded. To minimize stress and potential carryover effects, behavioral assays were conducted in a fixed order from less to more stressful, and animals were allowed to rest undisturbed in their home cages between tests. On Day 18, the all animals were submitted to the sucrose preference test. On Day 20, behavioral battery was performed in the following order: (I) open field test, (II) rotarod test, and (III) tail suspension test, with standardized rest 1 h intervals between assays. All apparatuses were cleaned with 70% ethanol between animals to reduce olfactory cues.

### Sucrose preference test

On the 18th day, the animals were presented with two water bottles on opposite sides of the cage, one containing only water and the other containing a 2% sucrose solution, for a 24-h habituation period. After this period, the animals were separated individually and again supplied with two previously weighed bottles with the same distribution for another 24 h. During this period, the bottles were switched sides once to avoid the animal’s tendency towards one of the bottles. At the end of this period, the bottles were weighed again, and consumption was established as the difference in bottle weights between before and after the 24-h period (before–after). The percentage of sucrose consumption was calculated using the equation: % preference for sucrose = sucrose consumption × 100/total consumption. Total consumption was defined as the sum of water consumption and sucrose consumption.

### Open field test

The open field test also represents a motor assessment of the animal. This test consists of a square box (45 cm × 45 cm) with 40 cm high walls that prevent the animal from escaping. The floor of the boxes was marked with a grid of lines (separated by 9 cm), and the mice were placed individually in one of the corners of the field. The number of lines crossed during a 5-min test session was recorded and counted as the total distance (Liu et al. 2015). The open field test was performed on the 20th day of the experiment.

### Rotarod test

The rotating bar test, or rotarod, represents one of the leading and oldest approaches to characterizing motor dysfunctions in animal models. It is a test that evaluates the motor coordination and balance of the animal by its ability to remain on the cylindrical rod that moves rotationally at a constant or accelerated speed for a certain period. The protocol used in this study was based on the work of (Liu et al. 2015), with some adaptations. The apparatus consists of four animal dividers and a cylindrical bar. The animals were initially habituated to the rotating bar for 5 min the day before the test at a constant speed of 5 rpm. On the 20th day, the animals were previously habituated for 60 min in the room of the rotarod test. They were placed on the rotating rod, and the protocol was performed in the accelerated mode of the apparatus’s speed levels (5 to 37 rpm). The animals remained on the rod for a maximum period of 300 s, during which time the latency to fall was recorded, which reflected the level of motor impairment of the animal (Liu et al. 2015).

### Tail suspension test

The tail suspension test consists of evaluating “despair-like” behavior, which represents an endophenotype of induced depression in animals. In this test, animals are suspended by their tails with adhesive tape in a position they cannot escape or hold onto nearby surfaces; the lack of escape-related behavior is considered immobility (Can et al. 2012). We adopted the protocol by Can et al. (2012) with modifications, in which the animals were individually suspended by their tails and accustomed to that position for one minute, then remained in the same position for another 5 min, where the immobility time was recorded by an observer 1 m away. After this total period of 6 min, the animals were returned to their respective boxes. The tail suspension test was performed on the 20th day.

### Immunofluorescence

The sections of frozen proximal colon samples (3 animals per group) were incubated with primary antibodies: occludin (Santa Cruz, 1:200) and GPR43 (Bioss antibodies, bs13536R, 1:50) in antibody dilution buffer (1 × PBS/1% BSA/0.3% Triton X-100).

Substantia nigra (3 animals per group) sections were incubated for double labeling with the primary antibodies anti-tyrosine hydroxylase (Invitrogen OPA1-0405, 1:500), p-CREB (Cell Signaling, #9198, dilution 1:500), BDNF (Alomone Labs, ANT-010, dilution 1:300), IBA-1 (Wako, 019-19741, dilution 1:500) and GFAP (Invitrogen, 130,300, dilution 1:1000). Later, the slides were left at 4 °C under constant and mild agitation, overnight. After approximately 16 h, the primary antibodies were removed, the sections were washed with TBS-T 1X solution and then incubated with conjugated secondary antibody fluor 488 anti-rabbit (Invitrogen A11008, dilution 1:100) or conjugated secondary antibody fluor 546 anti-mouse (Invitrogen A11003, 1:100) for 1 h at room temperature, and subsequently, incubated with 6-diamidino-2-phenylindole (DAPI, Invitrogen, 1:200) for 5 min in the dark. The slices were washed, mounted in ProLong Gold Antifade (Invitrogen™ P36930), observed under a fluorescence microscope Leica DMI8 system, and processed with the Leica Application Suite LAS software (Leica Microsystems, Wetzlar, Germany). Figures were exported as tiff files with Adobe Photoshop version 8. The protein immunoreactivity was quantified in several images for each mouse. Cell body counting and pixel density were quantified using the Gimp 2.10.18 program (GNU Image Manipulation Program, UNIX platforms).

### Quantification of serotonin and butyrate levels

Serotonin levels in brain supernatant were assessed through enzyme-linked immunosorbent assays (ELISA) using specific commercial kit (Elabscience® E-EL-0033). Serum and brain butyrate levels were measured using specific commercial kit (CEO777Ge, Cloud-clone, Wuhan, China). All procedures were performed in duplicate in accordance with the manufacturers’ instructions.

### Western blot

The Substantia nigra, pre-frontal cortex and colon were rapidly dissected and homogenized in an extraction solution containing protease inhibitor cocktail (10 mM EDTA, Amresco, Solon, USA; 2 mM phenylmethane sulfonyl fluoride, 100 mM NaF, 10 mM sodium pyrophosphate, 10 mM NaVO4, 10 µg of aprotinin / mL and 100 mM Tris, pH 7.4—Sigma–Aldrich). The samples (10 animals/per group) were mixed and homogenized to form a pool for each group. The homogenates were centrifuged and frozen at − 80 °C. The proteins (30 μg/μL) were separated on sodium dodecyl sulfate (SDS) polyacrylamide by gel electrophoresis under reduced conditions and were then transferred to the nitrocellulose membrane (THERMO SCIENTIFIC 88018) by Mini-PROTEAN® precast gels (BIO-RAD 1658004). After, the membranes were blocked with 3% BSA and incubated overnight with the following antibodies diluted in blocking solution (1.5% BSA, 0.02% Tris phosphate-buffered, and 0.01% Tween):

- Substantia nigra and pre-frontal cortex: tyrosine hydroxylase (Merck AB152, 1:500), α-synuclein (Santa Cruz, 1:1000), IBA-1 (Wako 016-200001, 1:500), phospho-α synuclein (Abcam, ab51253), anti IL-1β (Genway GWP-BPB232, 1:1000), anti-p-NFkB (Abcam, ab97726, 1:1000), p-CREB (Cell signaling #9198, 1:1000), anti-iNOS (BD 610600, 1:1000) and BDNF (Alomone ANT-10, 1,500), PSD-95 (Alomone #APZ-009, 1:500), serotonin transporter-SERT (Alomone labs AMT004, 1:200), GPR109 (Invitrogen PA5-90,579, 1:500).
- Colon: ocludin (Santa cruz sc-133256, 1:500); zonula occludens (Santa cruz—sc-33725, 1:500); phospho-α synuclein (Abcam ab51253, 1:500); phospho-NFkB (Cell signalling #3003, 1:1000); IL-1β (abcan ab9722, 1:1000).

After washing, the membranes were incubated with peroxidase-conjugated anti-rabbit horseradish (HRP) (Sigma-Aldrich A9169, 1:8000), and anti-mouse HRP (Sigma-Aldrich A0161, 1:5000). A chemiluminescence reagent (Super Signal, Pierce, Ref. 34,080) was used to make the labeled protein bands visible in the iBright CL 1000 system (Thermo Fisher Scientific, A44241). Later, the protein antibodies were stripped from the membranes, which were reprobed with the monoclonal anti-β-actin antibody (Sigma-Aldrich A2228, 1: 5000) as a loading control. Protein densitometry was performed using the Image J 1.38 software (NIH, MD, USA). The results were confirmed by at least three repetitions for each protein investigated, and statistical analyses were performed using the values that were obtained.

#### 16S rRNA gene sequence-based microbiota analysis

After euthanasia, fecal samples (5 mice per group) were collected and immediately put at 4 °C (on ice) and subsequently stored at − 80 °C. For the metagenomic sequencing, the DNA was extracted using magnetic beads (Christoff et al., 2017). Sequencing library preparation for bacterial identification was prepared using the V3/V4 16S rRNA gene 341F (CCTACGGGRSGCAGCAG) (Wang and Qian 2009) and 806R (GGACTACHVGGGTWTCTAAT) (Caporaso et al. 2012) primers, targeting an approximately 465 bp amplicon, and the samples were sequenced in a MiSeq system (Illumina, USA), using the standard Illumina primers provided by the manufacturer kit. Raw data from MiSeq was processed using a pipeline owned by Neoprospecta company. Illumina FASTQ files had the primers trimmed and their accumulated error evaluated (Phred < 20, equivalent to a maximum of 1% accumulated error across bases). Besides, clusters with abundances lower than 2 were removed (in some protocols, this threshold is set at < 5 to further reduce noise). Taxonomic ranks were allocated using a proprietary 16S rRNA sequence database (NeoRef, Neoprospecta Microbiome Technologies, Brazil) set at a 99% identity level using blastn v.2.6.0 + (Altschul et al., 1990). The resulting oligotype tables, analogous to traditional OTU tables, were used for calculating α-diversity metrics using R (version 4.1.0) and the Phyloseq package, with normalization via rarefaction to a standardized sequencing depth (e.g., 10,000 sequences per sample) or cumulative sum scaling (CSS) to account for differences in library sizes. Relative abundance plots were made in R (version 4.1.0). Regarding species-level resolution limits, the V3-V4 region enables reliable genus-level classification and species-level assignment for many taxa at 99% sequence identity; however, it may not distinguish closely related species in all cases due to conserved sequences in this ~ 465 bp amplicon, with approximately 17% of sequences potentially unclassifiable to species level in complex samples.

#### Statistical analysis

The analysis of the diversity of the gut microbiota was computed by the Hill diversity series (Hill., 1973), where q = 0 is the richness, q = 1 is the Shannon’s entropy, and q = 2 is the Reciprocal Simpson index. Beta diversity was analyzed using principal coordinate analysis (PCoA) to evaluate differences in taxonomic profiles between experimental groups. The PCoA was performed on log-ratio transformed values using Bray–curtis. The analyses were performed in R environment (R Core Team 2023), by the vegan package (Oksanen et al. 2020). The parametric data were analyzed by variance analysis (ANOVA one-way) followed by Tukey’s post-test or Holm-Sidak multiple comparisons tests. The data were represented by mean ± SD or mean ± confidence interval, and probability values lower than 0.05 were considered significant.

## Results

### Prebiotics improved rotenone-induced motor deficit

**Fig. 1:**
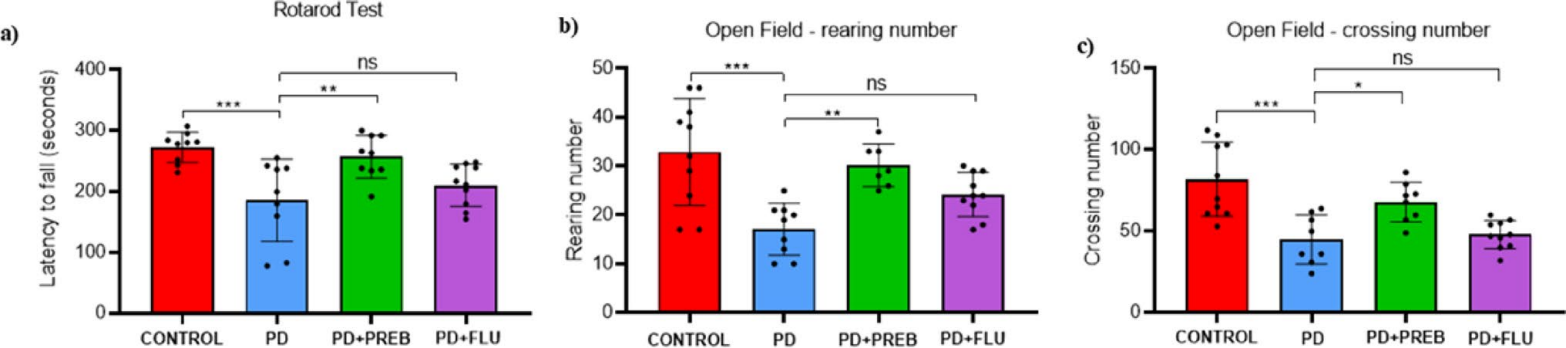
**a** Rotarod test (latency to fall): results expressed under the parameter of latency to fall from the rotating cylindrical platform in seconds (s); **b** Open field test (rearing number): results expressed under the parameters of rearing movements. **c** Open field test (crossing number): results expressed under the parameters of crossing movements; The data were analyzed by one-way ANOVA statistical tests of variance and Tukey’s post hoc test. NS: non-signifcant; *p < 0.05; **p < 0.01; ***p < 0.001 (*n* = 8–10 mice/group)

Animals induced by rotenone presented motor deficits observed in the rotarod test and open field tests. In the rotarod test, one-way ANOVA showed statistically significant differences among the experimental groups (F (3, 34) = 7.587, *p* = 0.0005), the PD group presented a shorter latency time to fall from the rotating cylindrical platform (CONTROL vs PD, *p* = 0.0009, Fig. 1A), indicating a lower locomotion capacity than the CONTROL group. On the other hand, mice treated with FOS and GOS in the PD + PREB group presented a longer latency time than the PD group (PD + PREB vs PD, *p* = 0.0071, Fig. 1A), indicating a greater motor capacity. Fluoxetine did not attenuate the motor deficit in animals in the PD + FLU group (PD + FLU vs PD, *p* = 0.45, Fig. 1A).

Two parameters were evaluated in the open field test: 1. Number of lifts, and 2. Number of crossings. One-way ANOVA showed statistically significant differences among the experimental groups in terms number of lifts (F (3, 32) = 8.975, *p* = 0.0002) and number of crossings (F (3, 33) = 10.45, *p* < 0.0001). The number of rearings was reduced in the PD group compared to the CONTROL group (CONTROL vs. PD, *p* = 0.0002, Fig. 1B), demonstrating a loss in the natural exploratory behavior of the animals. The number of crossings was also reduced in the animals of the PD group compared to the CONTROL group (CONTROL vs PD, *p* = 0.0002, Fig. 1C), indicating that the animals also presented difficulty in locomotion. However, treatment with prebiotics increased the number of rearings (PD + PREB vs. PD, *p* = 0.0047, Fig. 1B) and the number of crossings (PD + PREB vs. PD, *p* = 0.0349, Fig. 1C) in the PD + PREB group compared to the PD group, indicating an improvement in exploratory behavior and attenuation of bradykinesia. The group of animals treated with fluoxetine showed no improvement in the number of lifts (PD + FLU vs PD, *p* = 0.1485, Fig. 1B) or the number of crossings (PD + FLU vs PD, *p* = 0.8941, Fig. 1C).

### Prebiotics improved rotenone-induced depressive-like behavior

**Fig. 2:**
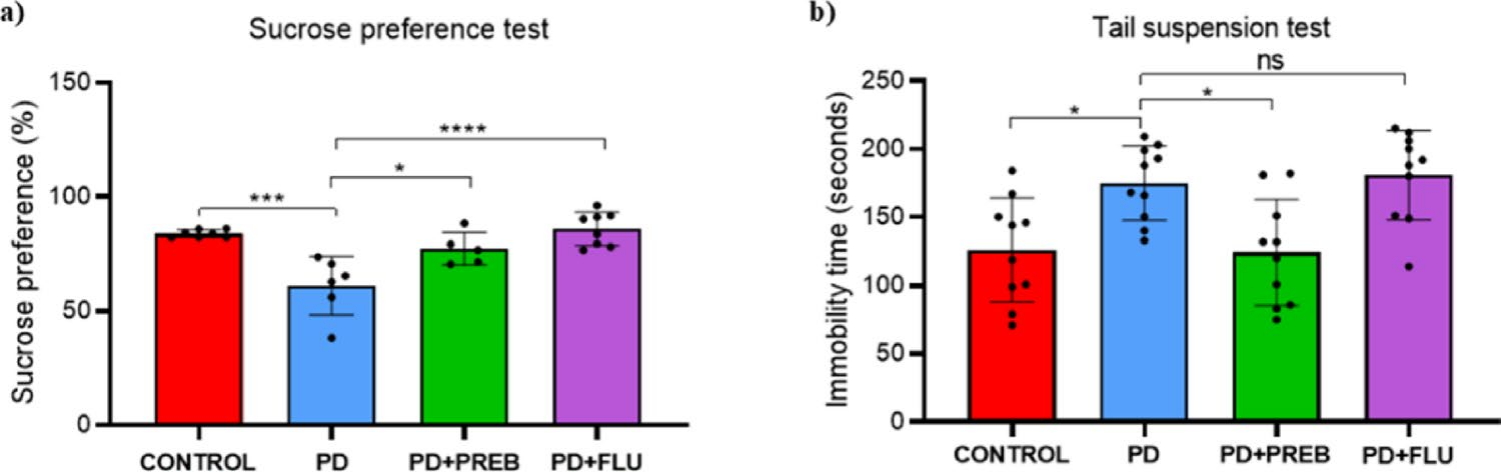
**a** Sucrose preference test: results expressed under the parameter of the animal’s preference for water with sucrose in percentage (%); **b** Tail suspension test: results expressed under the parameter of the immobility time of the depressed animal in seconds (s). The data were analyzed by one-way ANOVA statistical tests of variance and Tukey’s post hoc test. NS: non-signifcant; *p < 0.05; ***p < 0.001; ****p < 0.0001 (*n* = 5–10 mice/group)

Animals were evaluated for depressive-like behavior by sucrose preference and tail suspension tests, one-way ANOVA showed statistically significant differences among the experimental groups (F (3, 22) = 12.58, *p* < 0.0001) and (F (3, 36) = 7. 794, *p* = 0.0004), respectively. In the sucrose preference test, animals induced with rotenone showed a sucrose preference of less than 60%, while animals in the CONTROL group showed a sucrose preference above 80%. This lower preference in animals in the PD group demonstrates an anhedonic behavior (another endophenotype of depression) of these animals compared to animals in the CONTROL group (PD vs. CONTROL, *p* = 0.0002, Fig. 2A). In animals treated with prebiotics, sucrose preference was significantly higher compared to the PD group (PD + PREB vs. PD, *p* = 0.0154, Fig. 2A), meaning that treatment with prebiotics resulted in maintenance of the animal’s natural and healthy behavior of preferring the sugary liquid, with preference levels similar to those presented by the control group (> 95%). As expected, treatment of sick animals treated with fluoxetine also showed an improvement in sucrose preference (PD + FLU vs. PD, *p* < 0.0001, Fig. 2A).

Regarding the tail suspension test, rotenone-induced animals showed a longer immobility time compared to healthy animals (PD vs. CONTROL, *p* = 0.0159, Fig. 2B), indicating the development of despair-like behavior (an endophenotype of depressive symptoms). In animals treated with prebiotics, the immobility time was significantly reduced, indicating improvement of depressive symptoms (PD + PREB vs. PD, *p* = 0.0120, Fig. 2B). Conversely, the rotenone-induced and fluoxetine-treated group showed no significant improvement in immobility compared to the rotenone-induced group alone (PD + FLU vs. PD, *p* = 0.9817, Fig. 2B).

### Prebiotics increased serotonin and butyrate levels

**Fig. 3:**
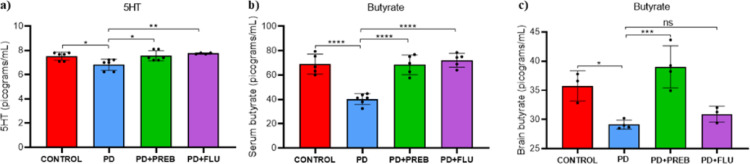
**a** Brain serotonin (5HT) levels; **b** Butyrate serum levels; and **c** brain butyrate levels. The data were analyzed by one-way ANOVA statistical tests of variance and Tukey’s post hoc test. NS: non-significant; *p < 0.05; **p < 0.01; ***p < 0.001; ****p < 0.0001 (*n* = 3–6 mice/group)

One-way ANOVA showed that there were statistically significant differences among the experimental groups regarding to the levels of serotonin (F (3, 18) = 6.583, *p* = 0.0034), serum and brain butyrate (F (3, 17) = 28.18, *p* < 0.0001) and (F (3, 10) = 13.49, *p* = 0.0008), respectively (Fig. 3). Rotenone induction promoted a decrease in brain serotonin levels compared to healthy animals (PD vs. CONTROL, *p* = 0.0202, Fig. 3A). In contrast, the serotonin levels significantly increased in the animals treated with prebiotics (PD + PREB vs. PD, *p* = 0.0137, Fig. 3A). As expected, fluoxetine also increased brain serotonin levels in the PD + FLU group (PD + FLU vs PD, *p* = 0.0059, Fig. 3A).

PD group showed reduced serum and brain butyrate levels compared to the CONTROL group (PD vs. CONTROL, *p* < 0.0001, Fig. 3B; PD vs. CONTROL, *p* = 0.0205, Fig. 3C, respectively). However, treatment with FOS + GOS increased significantly serum and brain butyrate levels (PD + PREB vs. PD, *p* < 0.0001, Fig. 3B; PD + PREB vs. PD, *p* = 0.0008, Fig. 3C, respectively). Similarly, treatment with fluoxetine promoted an increase in the serum butyrate levels (PD + FLU vs PD, *p* < 0.0001, Fig. 3B).

### Prebiotics increased groups of bacteria producing short-chain fatty acids

**Fig. 4:**
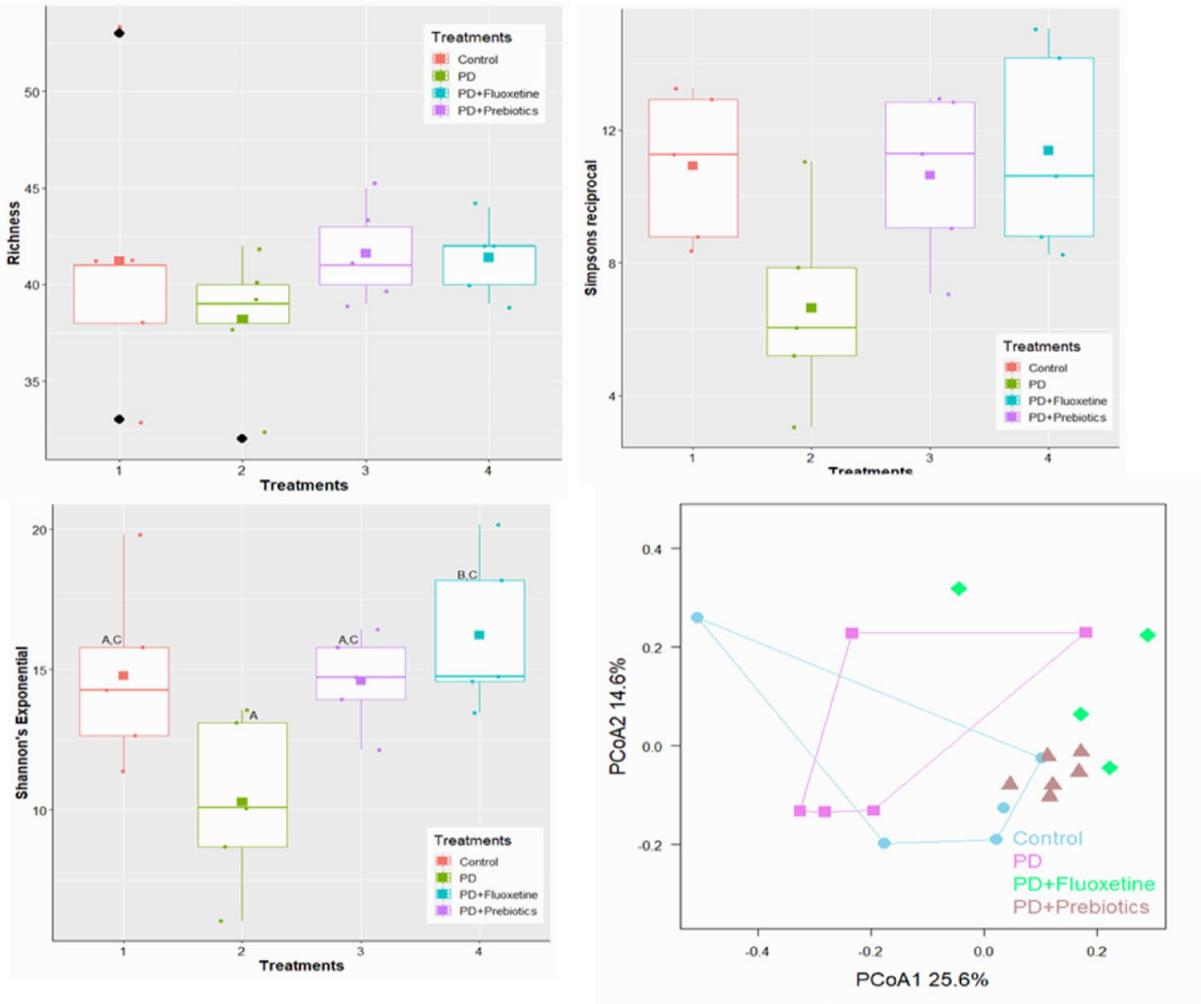
Detrimental changes in the gut microbiota diversity in rotenone-induce animals. Alpha diversity: Richness, Reciprocal Simpson index, and Shannon’s entropy index. Principal Coordinates analysis (PCoA). Alpha diversity indexes were determined by ANOVA one-way followed by Tukey’s post hoc test. Values are presented as mean ± SD (*n* = 5 cecal content/group). Different letters mean significant statistical difference (*p* < 0.01)

Bacterial DNA present in feces was analyzed through metagenomic sequencing analyses. The first data we can extract from the sequencing and identification of bacteria are related to bacterial communities’ α and beta diversity; there was a clear trend towards a decrease in diversity in the group subjected to PD and an increase in the treated groups, although without significance (Fig. 4).

**Fig. 5:**
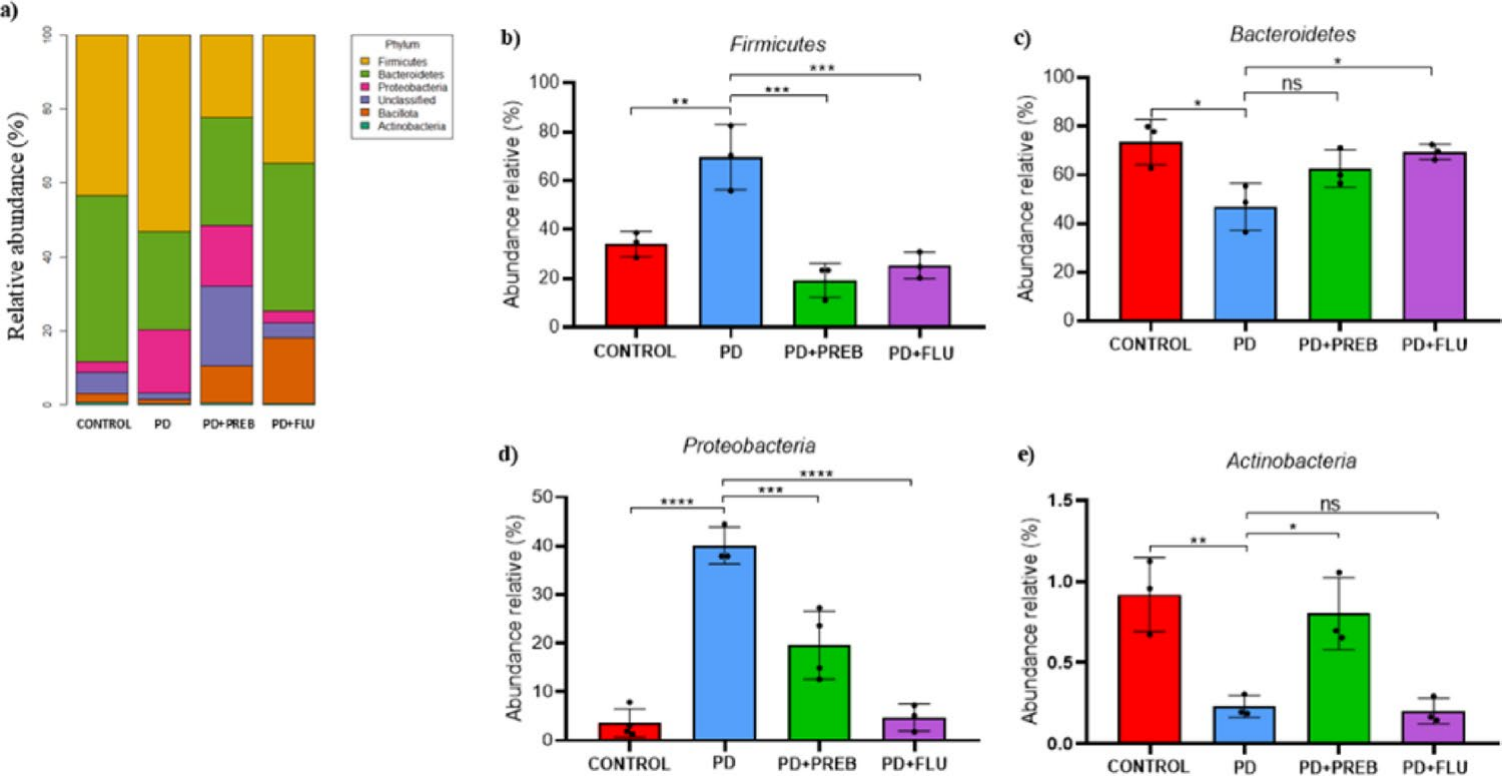
Sequencing of DNA bacterial 16 s gene in feces. **a** Distribution of relative abundance among phyla: **b** *Firmicutes*; **c** *Bacteroidetes*; **d** *Proteobacteria*; **e** *Actinobacteria*. The data were analyzed by one-way ANOVA–statistical tests of variance and tukey’s post hoc test. NS: non-significant; *p < 0.05; **p < 0.01; ***p < 0.001; ****p < 0.0001 (*n* = 5 mice/group)

One-way ANOVA showed statistically significant differences among the experimental groups about phyla *Firmicutes* (F (3, 30) = 8.422, *p* = 0.0003), *Bacteroidetes* (F (3, 9) = 5.910, *p* = 0.0164), *Proteobacteria* (F (3, 30) = 5.966, *p* = 0.0026) and *Actinobacteria* (F (3, 10) = 11.85, *p* = 0.0012). PD group showed an increase in the relative abundance of the phyla *Firmicutes* (PD vs. CONTROL, *p* = 0.0252, Fig. 5B) and *Proteobacteria* (PD vs. CONTROL, *p* = 0.0098, Fig. 5D) in the PD group compared to the CONTROL group. In contrast, PD group presented a reduced relative abundance of *Bacteroidetes* (PD vs. CONTROL, *p* = 0.0159, Fig. 5B) and *Actinobacteria* (PD vs. CONTROL, *p* = 0.0068, Fig. 5E) compared to the CONTROL group. Treatment with FOS and GOS promoted a reduction in the relative abundance of *Firmicutes* (PD + PREB vs. PD, *p* = 0.0004, Fig. 5B) and *Proteobacteria* (PD + PREB vs. PD, *p* = 0.0027, Fig. 5D) compared to the PD group. In contrast, prebiotic treatment increased the relative abundance of the *Actinobacteria* phylum (PD + PREB vs. PD, *p* = 0.0267, Fig. 5E). By its turn, fluoxetine treatment increased *Bacteroidetes* (PD + FLU vs. PD, *p* = 0.0312, Fig. 5B), whereas it reduced the relative abundance of *Firmicutes* (PD + PREB vs. PD, *p* = 0.0009, Fig. 5A), and *Proteobacteria* (PD + FLU vs. PD, *p* < 0.0001, Fig. 5D).

**Fig. 6:**
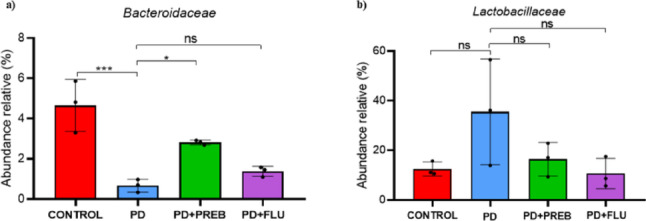
Sequencing of DNA bacterial 16 s gene in feces. Distribution of relative abundance of the **a** *Bacteroidaceae* and **b** Lactobacillaceae families. The data were analyzed by one-way ANOVA statistical tests of variance and Tukey’s post hoc test. NS: non-signifcant; *p < 0.05; ***p < 0.001 (*n* = 5 mice/group)

One-way ANOVA revealed statistically significant differences among the experimental groups about family *Bacteroidaceae* (F (3, 8) = 20.28, *p* = 0.0004). In the PD group, there was a decrease in the relative abundance of the *Bacteroidaceae* family (PD vs. CONTROL, *p* = 0.0004, Fig. 6A). Opposily, prebiotic treatment increased the relative abundance of both *Bacteroidaceae* (PD + PREB vs PD, *p* = 0.0196, Fig. 6A). In contrast, fluoxetine did not significantly alter the abundance of the family *Bacteroidaceae* (PD + FLU vs. PD, *p* = 0.5973, Fig. 6A). In relation to the *Lactobacillaceae* family, one-way ANOVA did not reach statistical significance (F (3, 8) = 2.878, *p* = 0.1031). In the PD group showed a trend to increase the relative abundance compared to the other group (Fig. 6B).

**Fig. 7:**
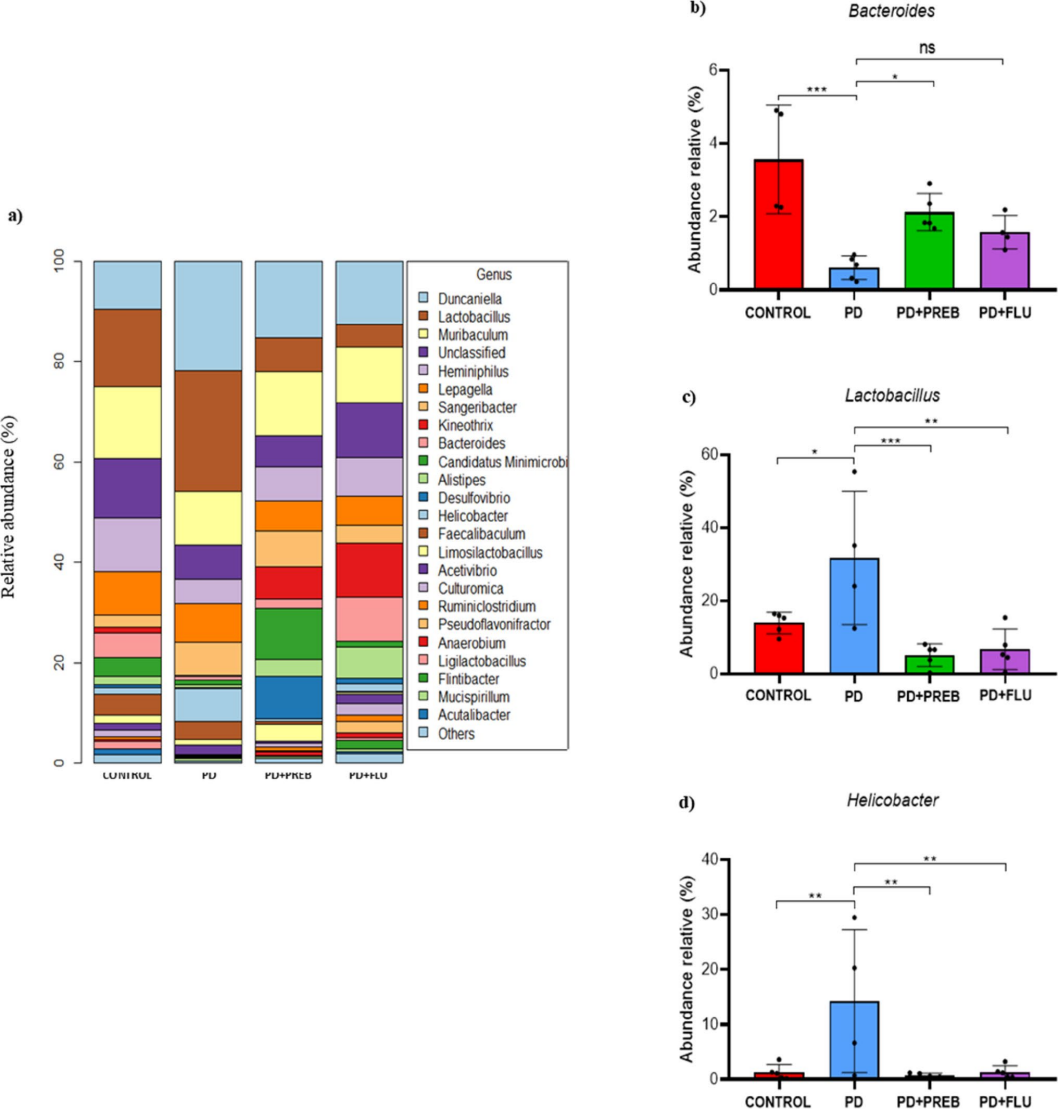
Sequencing of DNA bacterial 16 s gene in feces. **a** Distribution of relative abundance among genera: **b** *Bacteroides*; **c** *Lactobacillus*; **d** *Helicobacter*. The data were analyzed by one-way ANOVA statistical tests of variance and Tukey’s post hoc test. NS: non-signifcant; *p < 0.05; **p < 0.01; ***p < 0.001 (*n* = 5 mice/group)

Furthermore, one-way ANOVA revealed statistically significant differences among the experimental groups about genus *Bacteroides* (F (3, 14) = 10.73, *p* = 0.0006)*, Lactobacillus* (F (3, 17) = 9.636, *p* = 0.0006) *and Helicobacter* (F (3, 18) = 6.644, *p* = 0.0033), in the group of animals induced with rotenone, there was also a decrease in the relative abundance of the genus *Bacteroides* (PD vs. CONTROL, *p* = 0.0004, Fig. 7A and B) and an increase in the genera *Lactobacillus* (PD vs. CONTROL, *p* = 0.0186, Fig. 7A and C), and *Helicobacter* (PD vs. CONTROL, *p* = 0.0068, Fig. 7A and D), compared to the CONTROL group. In contrast, prebiotics increased the relative abundance of *Bacteroides* (PD + PREB vs. PD, *p* = 0.0398, Fig. 7A and B) and decreased that of the genera *Lactobacillus* (PD + PREB vs. PD, *p* = 0.0008, Fig. 7A and C) and *Helicobacter* (PD + PREB vs. PD, *p* = 0.0051, Fig. 7A and D). As with prebiotics, fluoxetine also reduced the abundance of the genera Lactobacillus (PD + FLU vs. PD, *p* = 0.0011, Fig. 7A and C) and Helicobacter (PD + FLU vs. PD, *p* = 0.0072, Fig. 7A and D) but did not significantly alter the genus Bacteroides (PD + FLU vs. PD, *p* = 0.3052, Fig. 7A and B).

**Fig. 8:**
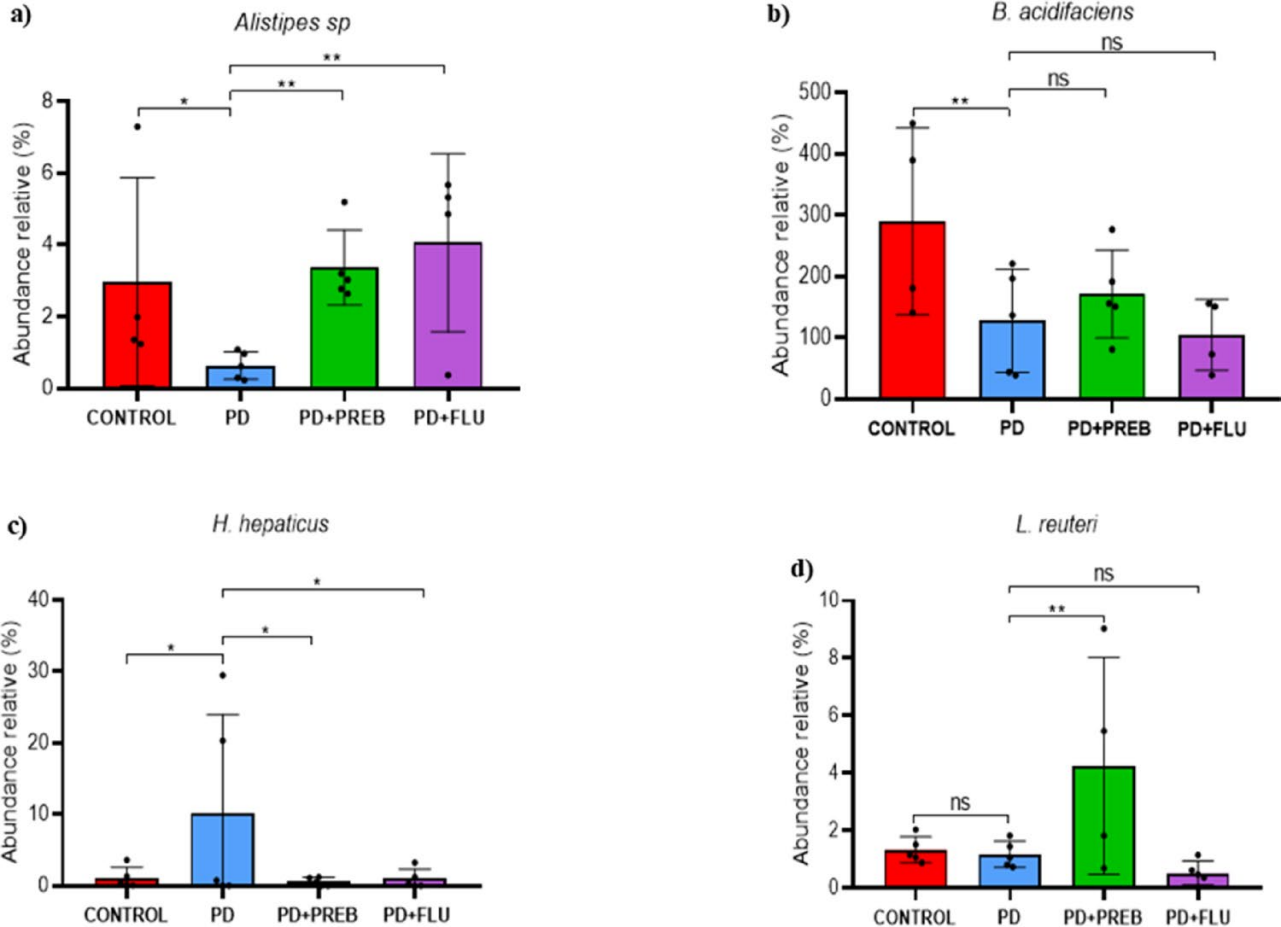
Sequencing of DNA bacterial 16 s gene in feces. **a** *Alistipes *sp.; **b** *B. acidifaciens*; **c** *Helicobacter hepaticus*; **d** Lactobacillus reuteri. The data were analyzed by one-way ANOVA statistical tests of variance and Tukey’s post hoc test. NS: non-signifcant; *p < 0.05; **p < 0.01; (*n* = 5 mice/group)

One-way ANOVA showed that there were statistically significant differences among the experimental groups regarding the *Alistipes ssp* (F (3, 32) = 6.820, *p* = 0.0011), *Bacteroides acidifaciens* (F (3, 32) = 6.973, *p* = 0.0010), *Helicobacter hepaticus* (F (3, 36) = 4.864, *p* = 0.0061) and *Lactobacillus reuteri* F (3, 34) = 8.779, *p* = 0.0002) relative abundance. We observed in the PD group decreased the relative abundance of *Alistipes ssp* (PD vs. CONTROL, *p* = 0.0391, Fig. 8A), *Bacteroides acidifaciens* (PD vs. CONTROL, *p* = 0.0030, Fig. 8B), and increased *Helicobacter hepaticus* (PD vs. CONTROL, *p* = 0.0220, Fig. 8C), but did not significantly alter the abundance of *Lactobacillus reuteri* (PD vs. CONTROL, *p* = 0.9965, Fig. 8D). However, prebiotic treatment significantly increased *Alistipes ssp* (PD + PREB vs PD, *p* = 0.0073, Fig. 8A), and *Lactobacillus reuteri* (PD + PREB vs. PD, *p* = 0.0019, Fig. 8D), but reduced *Helicobacter hepaticus* (PD + PREB vs PD, *p* = 0.0138, Fig. 8C). Fluoxetine increased the relative abundance of *Alistipes sp*. (PD + FLU vs. PD, *p* = 0.0013, Fig. 8A) and decreased *Helicobacter hepaticus* (PD + FLU vs. PD, *p* = 0.0194, Fig. 8C)*,* but did not affect *Bacteroides acidifaciens* (PD + FLU vs PD, *p* = 0.9493, Fig. 8B), and *Lactobacillus reuteri* (PD + FLU vs PD, *p* = 0.8053, Fig. 8D).

### Prebiotics increased immunoreactivity of GPR43 in the colon

**Fig. 9:**
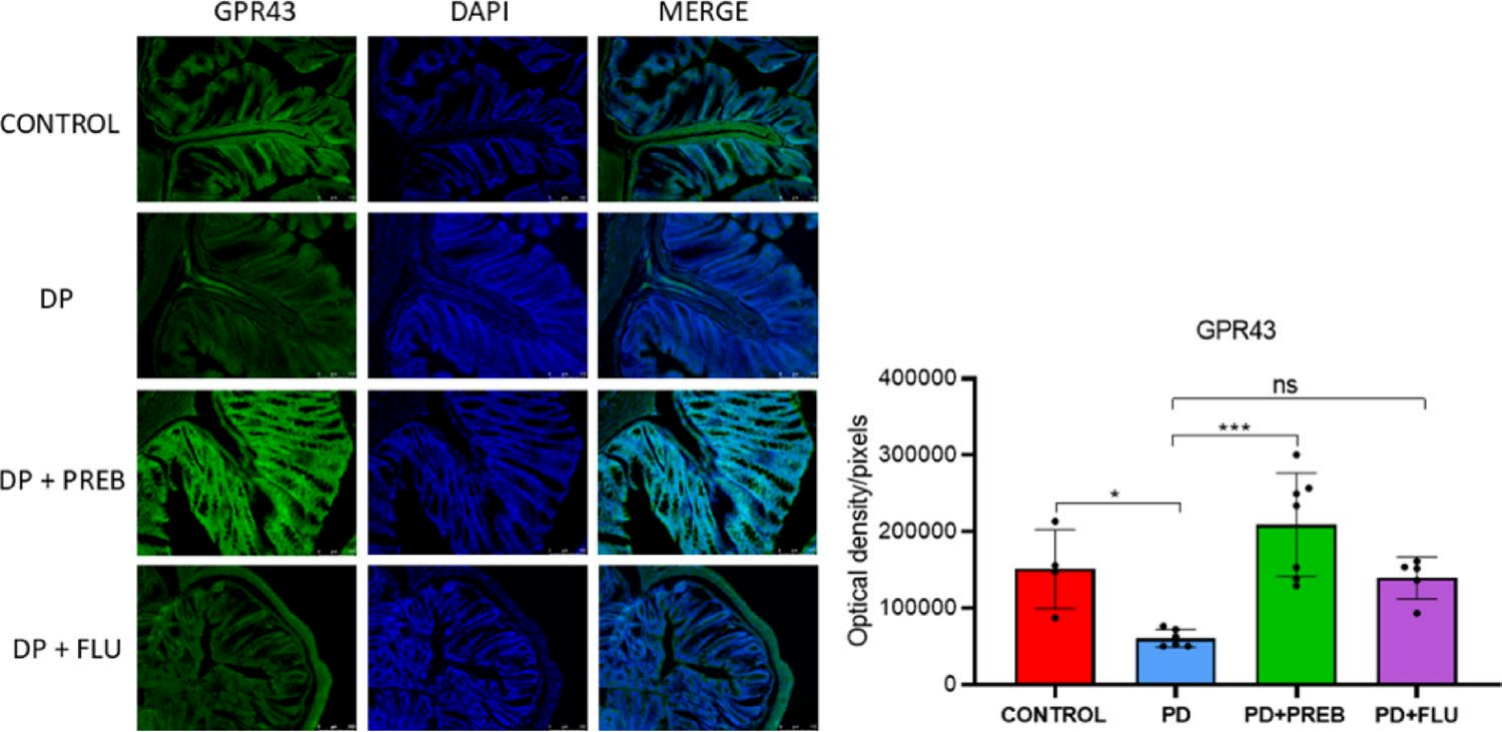
Representative images of immunofluorescence and optical density/pixels for GPR43 in the colon. The data were analyzed by one-way ANOVA statistical tests of variance and Tukey’s post hoc test (*n* = 4–7 images per group). NS: non-significant; *p < 0.05; ***p < 0.001

One-way ANOVA revealed statistically significant differences among the experimental groups about the expression of GPR43 (F (3, 18) = 10.92, *p* = 0.0003). In the colon, rotenone decreased the expression of GPR43, a receptor for short-chain fatty acids (PD vs. CONTROL, *p* = 0.0353). In contrast, prebiotics increased the expression of this receptor, indicating a greater responsiveness to short-chain fatty acids (PD + PREB vs. PD, *p* = 0.0001) (Fig. 9).

### Prebiotics reduced α-synuclein, inflammation and intestinal permeability in the colon

**Fig. 10:**
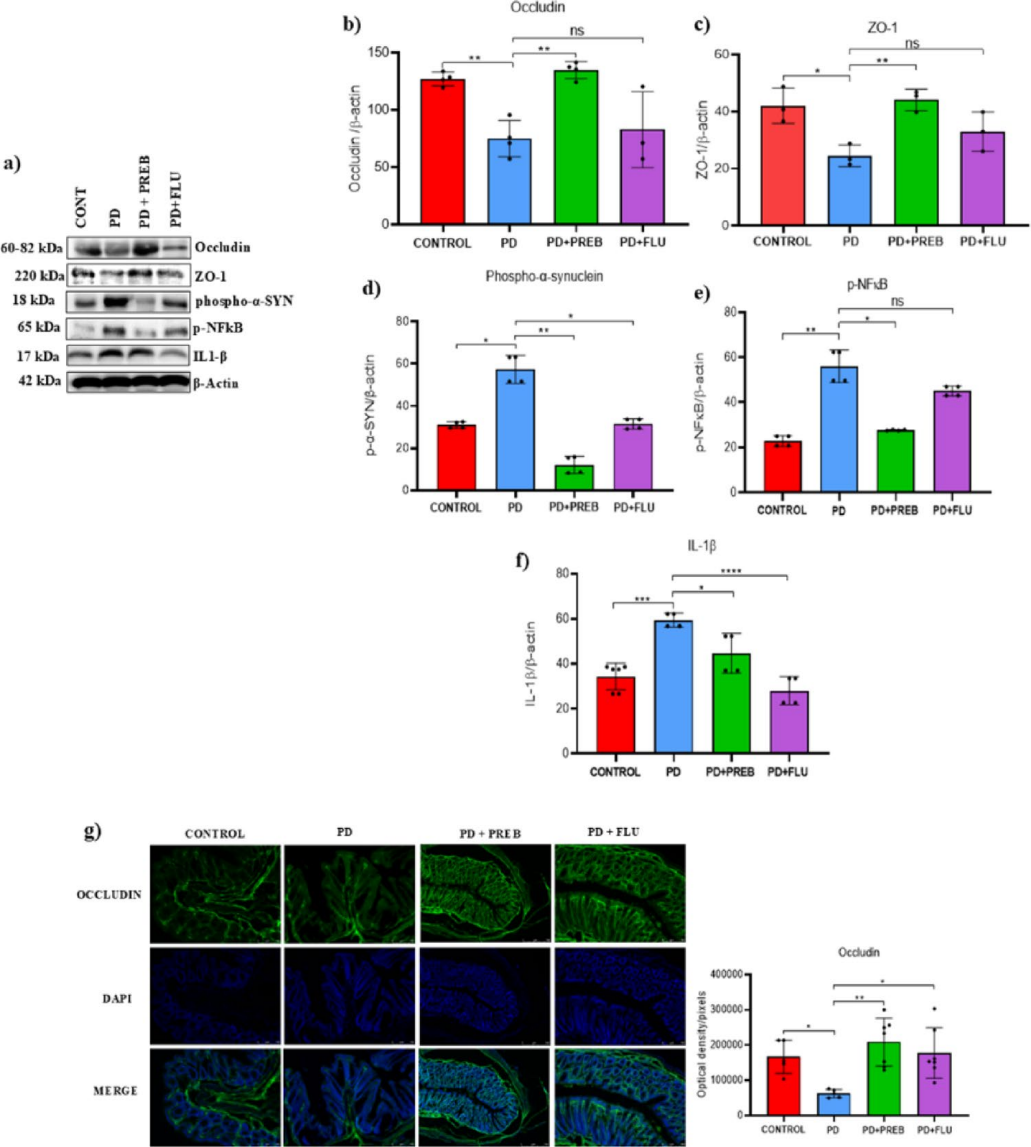
**a** Representative images of western blot bands (Left panels), and western blot quantification of **b** occludin, **c** zonula occludens, **d** phospho-α-synuclein, **e** NFkB, and **f** IL1-β in the colon. The data were analyzed by one-way ANOVA statistical tests of variance and Tukey’s post hoc test. NS: non-significant; *p < 0.05; **p < 0.01; ***p < 0.001; ****p < 0.0001; pooled samples of 10 mice/group; statistical analyses were performed using the values obtained in different replicates. **f** Immunofluorescence for occludin in the colon. The data were analyzed by one-way ANOVA statistical tests of variance and Tukey’s post hoc test. NS: non-signifcant; *p < 0.05; **p < 0.01 (*n* = 5–7 images/group)

One-way ANOVA showed that there were statistically significant differences among the experimental groups regarding to the protein levels of occludin (F (3, 11) = 11.98, *p* = 0.0009), zonula occludens (F (3, 8) = 8.538, *p* = 0.0071), α-synuclein (F (3, 4) = 27.40, *p* = 0.0040), p-NF-kB (F (3, 4) = 19.90, *p* = 0.0072) and IL1-β (F (3, 14) = 19.77, *p* < 0.0001) (Fig. 10A). We observed that the PD group showed a reduction in occludin and zonula occludens compared to the control group (PD vs. CONTROL, *p* = 0.058 and *p* = 0.0161, respectively, Fig. 10A–C), suggesting possible damage to the integrity of the epithelium in response to injury caused by rotenone. Moreover, rotenone induced-animals showed an accumulation of α-synuclein in the colon (PD vs. CONTROL, *p* = 0.0211, Fig. 10A and D), as well as an increase in p-NF-kB (PD vs. CONTROL, *p* = 0.0084, Fig. 10A and E), and IL1-β (PD vs. CONTROL, *p* = 0.0001, Fig. 10A and F). Prebiotics significantly increased occludin (PD + PREB vs PD, *p* = 0.0021, Fig. 10A and B), and zonula occludens (PD + PREB vs PD, *p* = 0.0087, Fig. 10A and C), while reducing the accumulation of α-synuclein (PD + PREB vs. PD, *p* = 0.0029, Fig. 10A and D), the expression of p-NF-kB (PD + PREB vs. PD, *p* = 0.0149, Fig. 10A and E), and IL1-β (PD + PREB vs. PD, *p* = 0.0234, Fig. 10A and F). Fluoxetine-treated group showed no significant the protein levels of occludin (PD + FLU vs. PD, *p* = 0 0.9276, Fig. 10A and B) and zonula occludens (PD + FLU vs. PD, *p* = 0.2814, Fig. 10A and C), however reduced the expression levels of phospho-α-synuclein (PD + FLU vs. PD, *p* = 0.0224, Fig. 10A and D), and IL1-β (PD + FLU vs. PD, *p* < 0.0001, Fig. 10A and F), but did not significantly reduce p-NF-kB (PD + FLU vs. PD, *p* = 0.2523, Fig. 10A and E).

The quantification of occludin was complemented by immunofluorescence analysis. One-way ANOVA revealed statistically significant differences among the experimental groups about the expression of occludin (F (3, 20) = 6.556, *p* = 0.0029) (Fig. 10G). Rotenone treatment significantly reduced the immunoreactivity to the junction protein occludin (PD vs. CONTROL, *p* = 0.0465, Fig. 10G). When compared to the PD group, the group that was induced with rotenone and treated with prebiotics showed an increase in occludin expression (PD + PREB vs. PD, *p* = 0.0019, Fig. 10G), as did the group treated with fluoxetine (PD + FLU vs. PD, *p* = 0.0145, Fig. 10G).

### Prebiotics reduced phosphorylated α-synuclein aggregation, glial reactivity and neuroinflammation in the substantia nigra

**Fig. 11:**
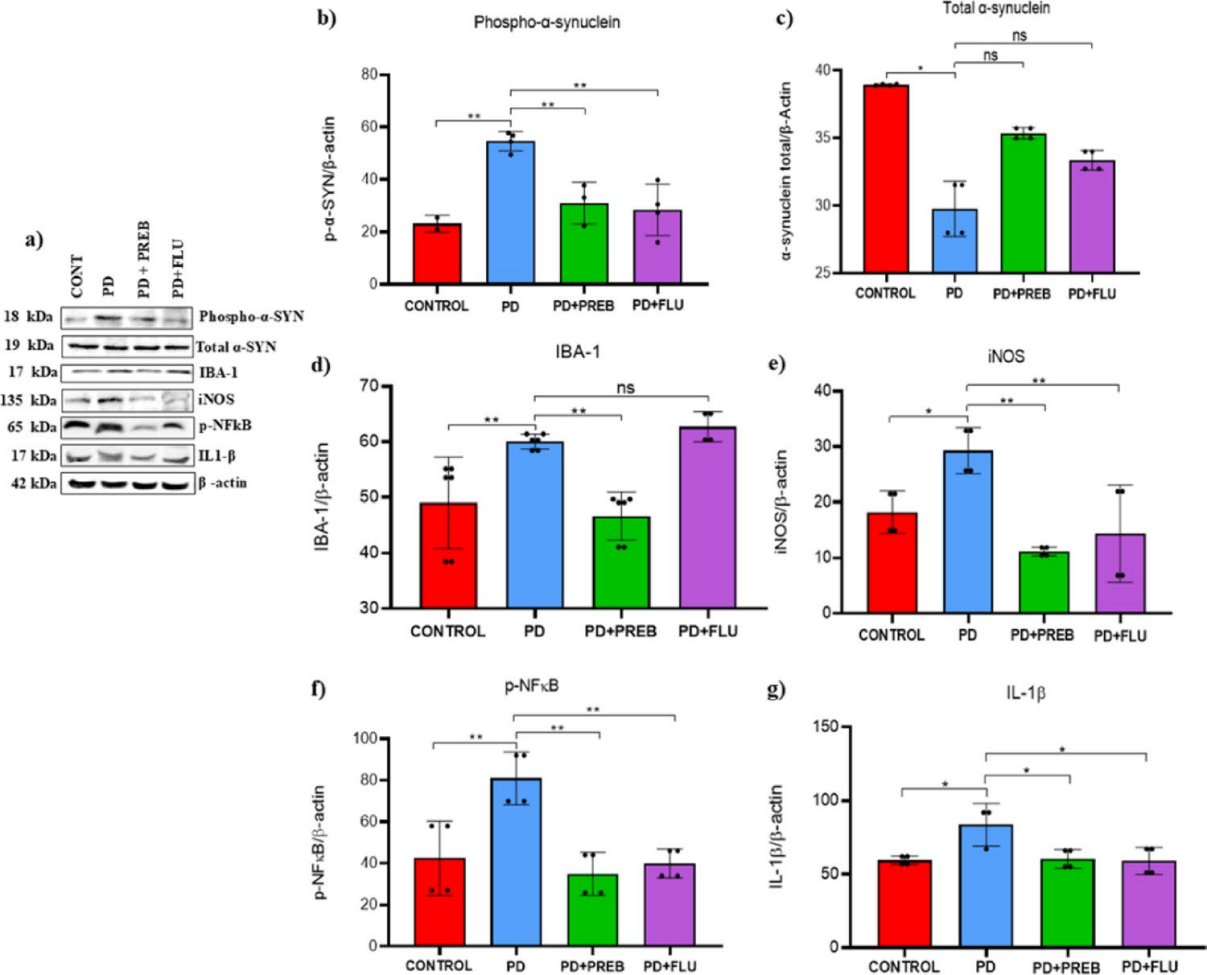
**a** Representative images of western blot bands (Left panels), and western blot quantification of **b** phospho-α-synuclein, **c** total α-synuclein; **d** IBA-1, **e** iNOS, **f** NF-kB and **g** IL1-β in the substantia nigra. The data were analyzed by one-way ANOVA statistical tests of variance and Tukey’s post hoc test. NS: non-signifcant; *p < 0.05; **p < 0.01; pooled samples of 10 mice/group; statistical analyses were performed using the values obtained in different replicates

To investigate the association between neuronal dysfunction associated with phosphorylated α-synuclein aggregation, we analyzed the expression of phospho-α-synuclein, total α-synuclein, IBA-1, iNOS, p-NFkB and IL1-β in the substantia nigra. One-way ANOVA revealed statistically significant differences among the experimental groups about the expression of phospho-α-synuclein (F (3, 9) = 12.96, *p* = 0.0013), total α-synuclein (F (3, 12) = 48.24, *p* < 0.0001), IBA-1 (F (3, 18) = 12.98, *p* < 0.0001), iNOS (F (3, 12) = 9.209, *p* = 0.0019), p-NFkB (F (3, 12) = 11.22, *p* = 0.0008) and IL1-β (F (3, 11) = 6.212, *p* = 0.0100). In the substantia nigra, rotenone induction caused an increase in phosphorylated α-synuclein in PD-induced animals (PD vs. CONTROL, *p* = 0.0032, Fig. 11 A and B) and the expression of IBA-1 (ionized calcium binding adaptor molecule) protein (PD vs. CONTROL, *p* = 0.0070, Fig. 11 A and D), indicating an increase in toxic protein aggregation and reactive microglia, respectively. In addition, PD group animals also showed an increase in nitric oxide synthase (iNOS) (PD vs. CONTROL, *p* = 0.0468, Fig. 11 A and E), p-NFkB (PD vs. CONTROL, *p* = 0.0048, Fig. 11 A and F), and IL1-β (PD vs. CONTROL, *p* = 0.0166, Fig. 11 A and G), which are mediators of inflammatory pathways. Prebiotic treatment reduced the expression of phosphorylated α-synuclein (PD + PREB vs. PD, *p* = 0.0090, Fig. 11 A and B) and attenuated microglial reactivity by reducing IBA-1 levels compared to the PD group (PD + PREB vs. PD, *p* = 0.0012, Fig. 11 A and D). Finally, prebiotics also reduced the expression of iNOS (PD + PREB vs PD, *p* = 0.0017, Fig. 11 A and E), p-NFkB (PD + PREB vs. PD, *p* = 0.0012, Fig. 11 A and F), and IL1-β (PD + PREB vs. PD, *p* = 0.0214, Fig. 11 A and G) in the substantia nigra. Fluoxetine also reduced phospho-α-synuclein levels (PD + FLU vs. PD, *p* = 0.0028, Fig. 11 A and B), iNOS (PD + FLU vs. PD, *p* = 0.0075, Fig. 11 A and E), p-NFkB (PD + FLU vs. PD, *p* = 0.0030, Fig. 11 A and F) and IL1-β (PD + FLU vs. PD, *p* = 0.0147, Fig. 11 A and G).

**Fig. 12:**
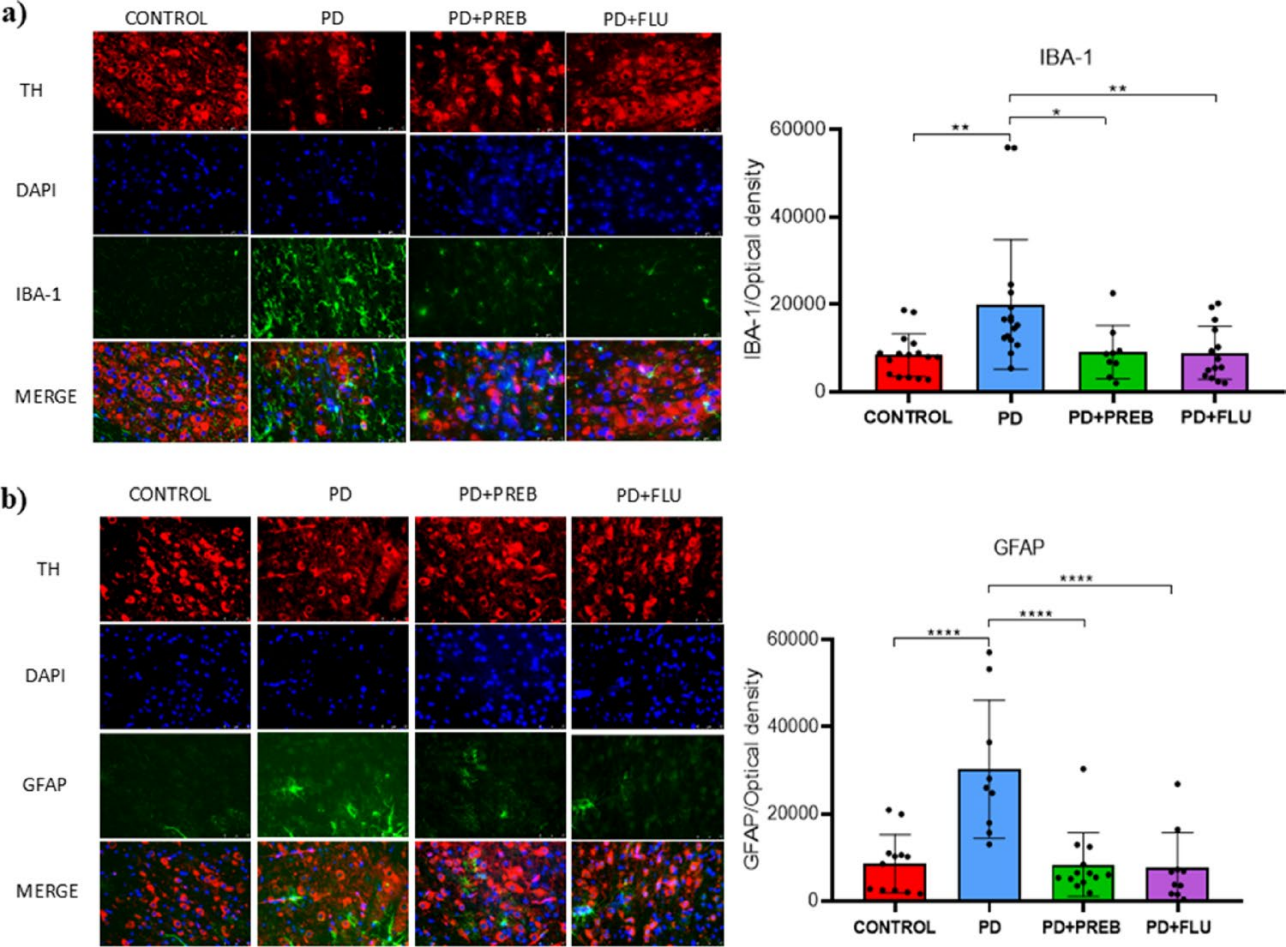
**a** Immunofluorescence for IBA-1 in the substantia nigra. The data were analyzed by one-way ANOVA statistical tests of variance and Tukey’s post hoc test. NS: non-significant; *p < 0.05; **p < 0.01 (*n* = 12 images/group); **b** Immunofluorescence for GFAP in the substantia nigra. The data were analyzed by one-way ANOVA statistical tests of variance and Tukey’s post hoc test. ****p < 0.0001. (*n* = 12 images/group)

Furthermore, the analysis of IBA-1 and GFAP expression was complemented by immunofluorescence assays. One-way ANOVA revealed statistically significant differences among the experimental groups about the expression of IBA-1 (F (3, 52) = 5.623, *p* = 0.0020) and GFAP (F (3, 40) = 12.44, *p* < 0.0001) (Fig. 12 A and B). Increased IBA-1 was also evidenced in the substantia nigra by immunofluorescence analyses in the rotenone-induced group (PD vs. CONTROL, *p* = 0.0047, Fig. 12A), while the prebiotic and fluoxetine-treated groups reduced IBA-1 labeling (PD + PREB vs. PD, *p* = 0.0327, Fig. 12A), (PD + FLU vs. PD, *p* = 0.0084, Fig. 12A). In addition, there was also an increase in glial fibrillary acidic protein (GFAP) in the PD group (PD vs. CONTROL, *p* < 0.0001, Fig. 12B). However, prebiotics and fluoxetine were able to attenuate the immunoreactivity of this glial marker (PD + PREB vs. PD, *p* < 0.0001, Fig. 12B), (PD + FLU vs PD, *p* < 0.0001, Fig. 12B).

### Prebiotics attenuated dopaminergic neuronal death and increased neuroplasticity factors in the substantia nigra

**Fig. 13:**
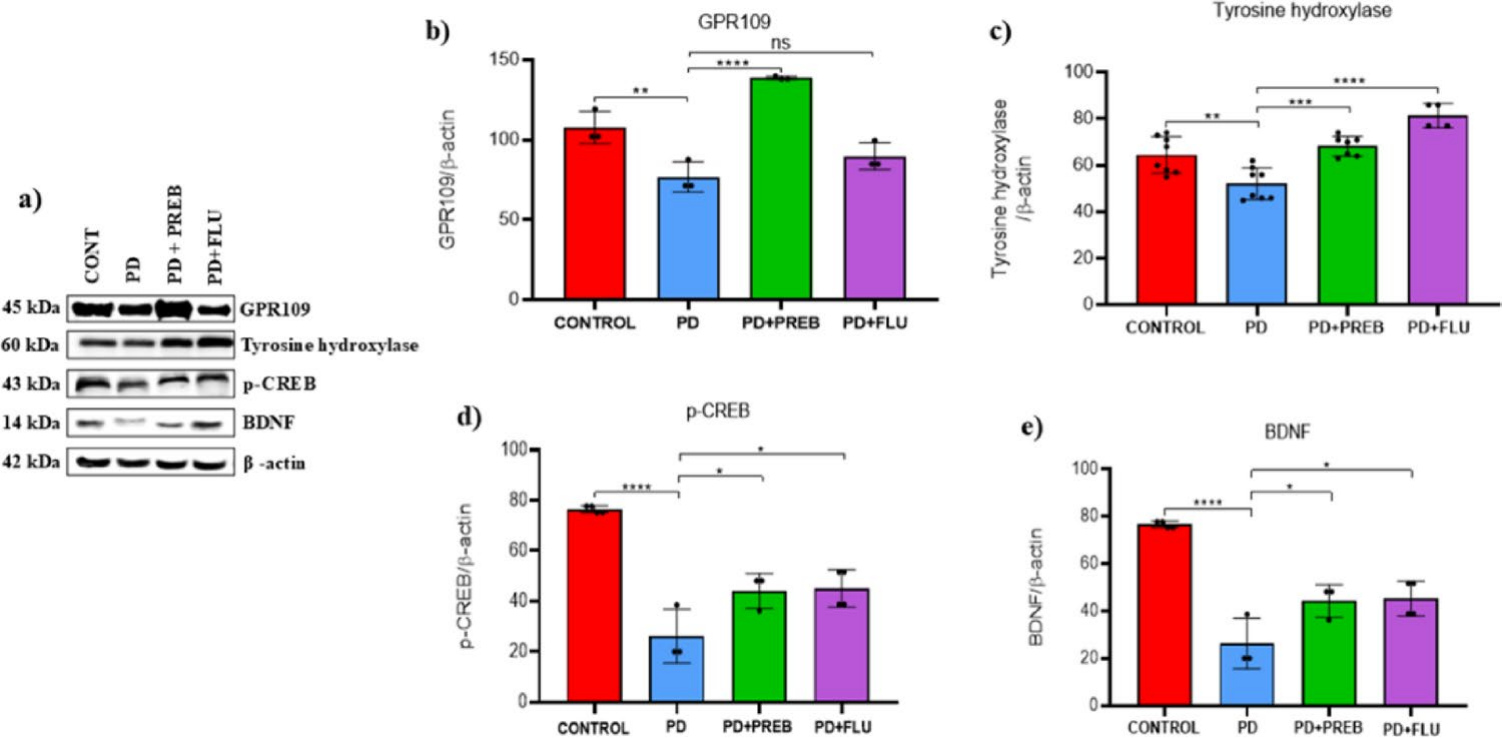
**a** Representative images of western blot bands (Left panels), and western blot quantification of **b** GPR109, **c** TH, **d** p-CREB and **e** BDNF in the substantia nigra. The data were analyzed by one-way ANOVA statistical tests of variance and Tukey’s post hoc test. NS: non-signifcant; *p < 0.05; **p < 0.01; ****p < 0.0001; pooled samples of 10 mice/group; statistical analyses were performed using the values obtained in different replicates

We employed western blot to evaluate the protein levels of the GPR109, enzyme tyrosine hydroxylase (TH), p-CREB and BDNF. One-way ANOVA showed statistically significant differences among the experimental groups regarding GPR109 (F (3, 8) = 32.73, *p* < 0.0001), TH F (3, 24) = 17.49, *p* < 0.0001), p-CREB (F (3, 10) = 31.68, *p* < 0.0001) and BDNF (F (3, 10) = 31.68, *p* < 0.0001) (Fig. 13B–E).

Analysis showed that systemic administration of rotenone promoted reduced GPR109 levels in the substantia nigra (PD vs. CONTROL, *p* = 0.0070, Fig. 13A and B). In contrast, treatment with prebiotics significantly increased the GPR109 levels (PD + PREB vs. PD, *p* < 0.0001, Fig. 13A and B).

The death of dopaminergic neurons in the substantia nigra was evidenced by decreased expression of the TH (PD vs. CONTROL, *p* = 0.0038, Fig. 13A and C). However, treatment with prebiotics was able to attenuate dopaminergic neuronal loss (PD + PREB vs. PD, *p* = 0.0007, Fig. 13A and C), just as fluoxetine (PD + FLU vs. PD, *p* < 0.0001, Fig. 13A and C). Rotenone caused loss of neuroplasticity through decreased p-CREB (PD vs. CONTROL, *p* < 0.0001, Fig. 13A and D) and BDNF (PD vs. CONTROL, *p* < 0.0001, Fig. 13A and E). However, prebiotics increased the protein expression of p-CREB (PD + PREB vs. PD, *p* = 0.0453, Fig. 13A and D) and BDNF (PD + PREB vs. PD, *p* = 0.0453, Fig. 13A and E). Fluoxetine also increased the expression levels of TH (PD + FLU vs. PD, *p* < 0.0001, Fig. 13A and C), p-CREB (PD + FLU vs. PD, *p* = 0.0237, Fig. 13A and D), and BDNF (PD + FLU vs. PD, *p* = 0.0237, Fig. 13A and E).

**Fig. 14:**
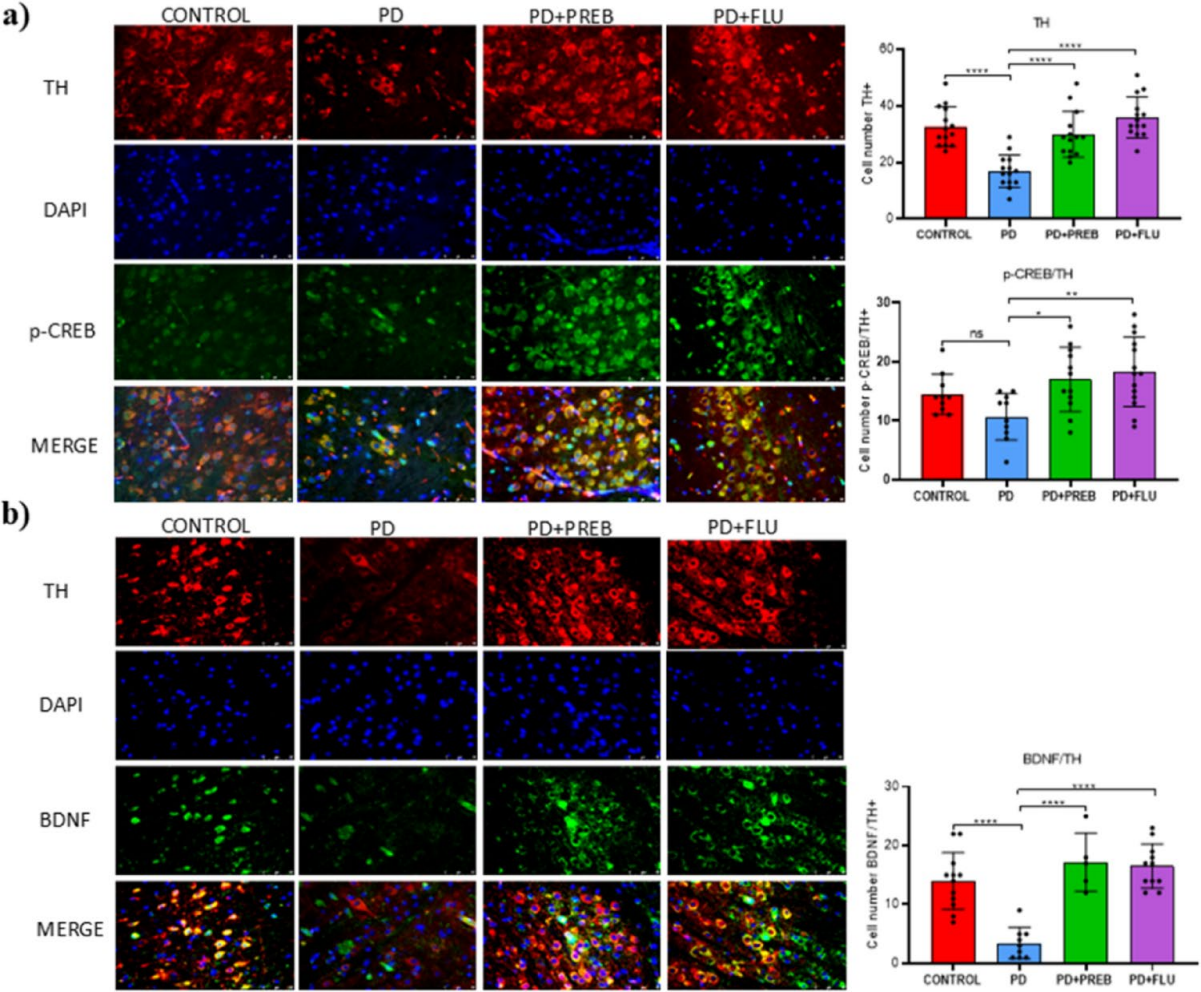
**a** Immunofluorescence for TH and pCREB in the substantia nigra. The data were analyzed by one-way ANOVA statistical tests of variance and Tukey’s post hoc test. NS: non-significant; *p < 0.05; **p < 0.01; ****p < 0.0001; (*n* = 12 images/group); **b** Immunofluorescence for TH and BDNF in the substantia nigra. The data were analyzed by one-way ANOVA statistical tests of variance and Tukey’s post hoc test. ****p < 0.0001. (*n* = 12 images/group)

Considering the essential role of the TH, p-CREB, and BDNF pathways in the plasticity and function of dopaminergic neurons, an immunofluorescence analysis was also performed to evaluate their expression in the substantia nigra. One-way ANOVA showed statistically significant differences among the experimental groups regarding the expression of TH (F (3, 52) = 19.25, *p* < 0.0001), p-CREB/TH (F (3, 42) = 5.134, *p* = 0.0041) and BDNF/TH (F (3, 33) = 21.11, *p* < 0.0001) (Figs. 14A and B). Analysis showed that administration of rotenone promoted reduced in TH immunoreactivity in the substantia nigra of the PD group (PD vs. CONTROL, *p* < 0.0001, Fig. 14A). Treatment with prebiotics increased TH immunostaining in the substantia nigra (PD + PREB vs PD, *p* < 0.0001, Fig. 14A), as fluoxetine (PD + FLU vs PD, *p* < 0.0001, Fig. 14A). Immunofluorescence analyses also confirmed the results of both p-CREB and BDNF by western blotting. The PD group showed a decrease in BDNF (PD vs. CONTROL, *p* < 0.0001, Fig. 14B) and p-CREB, although this latter was not statistically significant compared to the control (PD vs. CONTROL, *p* = 0.3265, Fig. 14A). In contrast, prebiotics increased BDNF (PD + PREB vs. PD, *p* < 0.0001, Fig. 14B), and p-CREB (PD + PREB vs. PD, *p* = 0.0239, Fig. 14A) labelling. Similarly, fluoxetine, the gold standard for neuroplastic responses, also increased both BDNF (PD + FLU vs. PD, *p* < 0.0001, Fig. 14B) and p-CREB (PD + FLU v.s PD, *p* = 0.0033, Fig. 14A). The overlap between BDNF and p-CREB labeling and dopaminergic (TH) labeling confirms the neuroplastic response of dopaminergic neurons after treatments.

### Prebiotics reduced rotenone-induced neuroinflammation in the prefrontal córtex

**Fig. 15:**
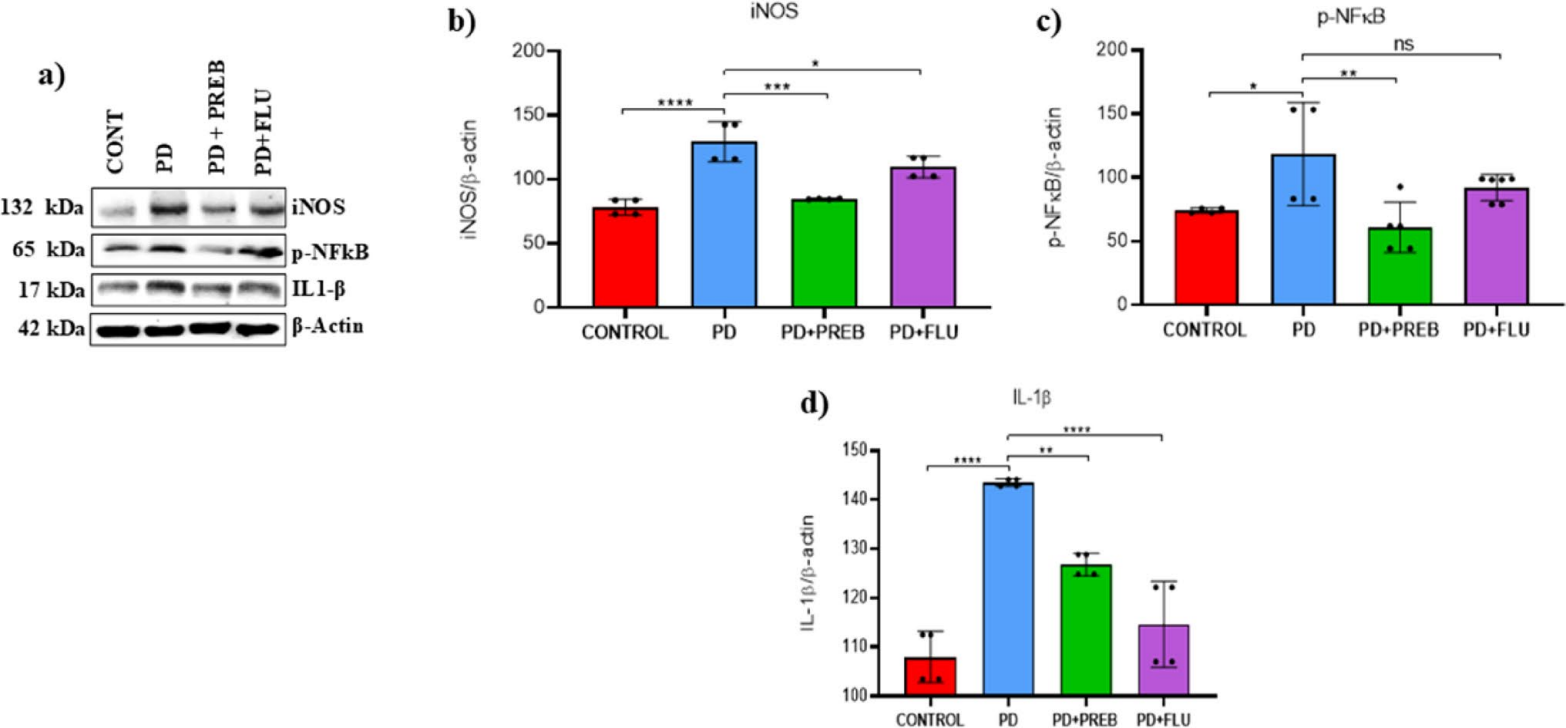
**a** Representative images of western blot bands (Left panels), and western blot quantification of **b** iNOS, **c** NF-kB and **d** IL1-β in the prefrontal córtex. The data were analyzed by one-way ANOVA statistical tests of variance and Tukey’s post hoc test. NS: non-signifcant; *p < 0.05; **p < 0.01; ***p < 0.001; ****p < 0.0001; pooled samples of 10 mice/group; statistical analyses were performed using the values obtained in different replicates

Given the involvement of cortical inflammation in Parkinson’s disease, we evaluated neuroinflammatory markers in the prefrontal cortex (PFC). One-way ANOVA showed statistically significant differences among the experimental groups regarding iNOS (F (3, 12) = 25.28, *p* < 0.0001), p- NFkB (F (3, 15) = 5.767, *p* = 0.0079) and IL1-β (F (3, 12) = 35.68, *p* < 0.0001) protein levels (Fig. 15B-D). The rotenone increased the expression of protein levels of iNOS (PD vs. CONTROL, *p* < 0.0001, Fig. 15A and B), phosphorylated NFkB (PD vs. CONTROL, *p* = 0.0496, Fig. 15A and C), and IL1-β (PD vs. CONTROL, *p* < 0.0001, Fig. 15A and D). However, in animals that were treated with prebiotics, there was a significant decrease in the expression levels of iNOS (PD + PREB vs. PD, *p* = 0.0001, Fig. 15A and B), p-NFkB (PD + PREB vs. PD, *p* = 0.0062, Fig. 15A and C), and IL1- β (PD + PREB vs. PD, *p* = 0.0034, Fig. 15A and D). Fluoxetine also decreased the expression levels of iNOS (PD + FLU vs PD, *p* = 0.0492, Fig. 15A and B), and IL1-β (PD + FLU vs. PD, *p* < 0.0001, Fig. 15A and D).

#### Prebiotics increased neuroplasticity in the prefrontal córtex

**Fig. 16:**
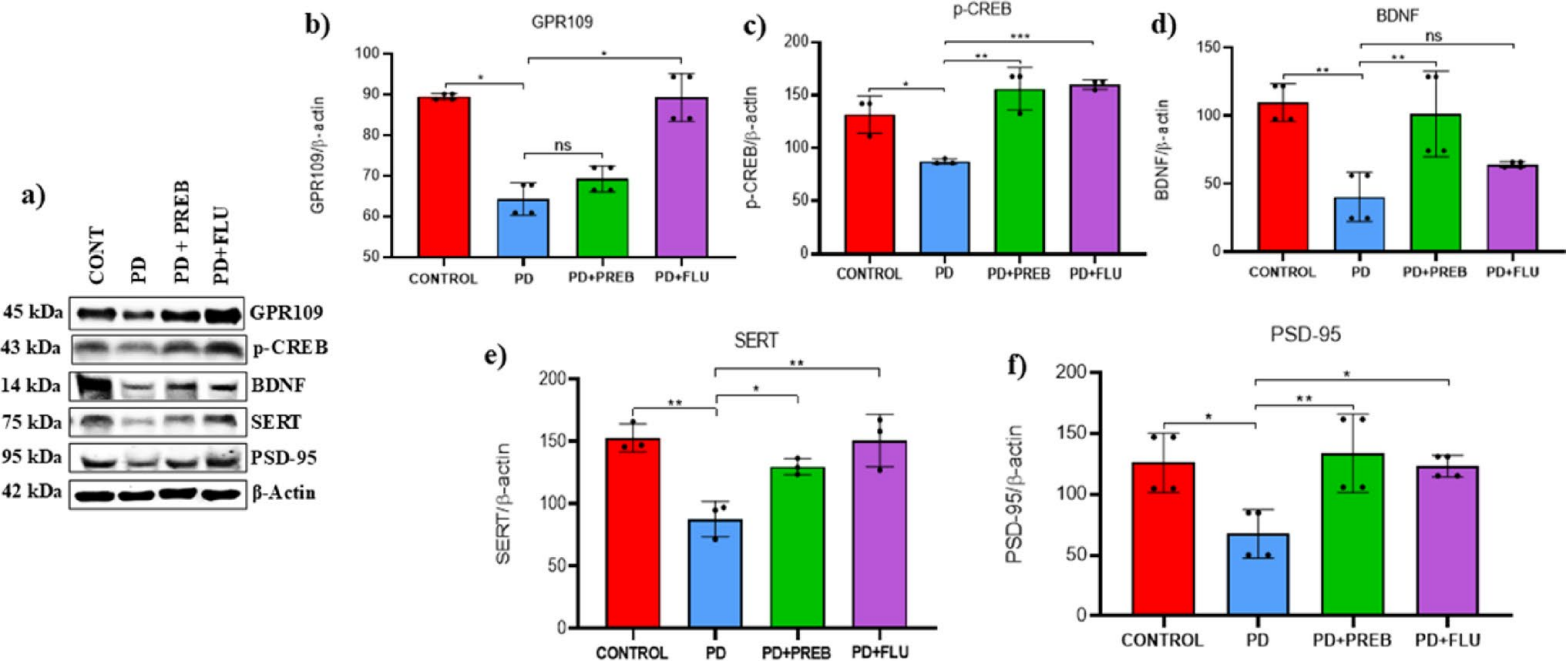
**a** Representative images of western blot bands (Left panels), and western blot quantification of **b** GPR109, **c** p-CREB, **d** BDNF, **e** SERT and **f** PSD-95 in the prefrontal córtex. The data were analyzed by one-way ANOVA statistical tests of variance and Tukey’s post hoc test. NS: non-signifcant; *p < 0.05; **p < 0.01; ***p < 0.001; pooled samples of 10 mice/group; statistical analyses were performed using the values obtained in different replicates

To determine neuroplasticity, we investigated the expression of GPR109, BDNF, p- CREB, SERT and PSD-95 in the prefrontal cortex (PFC). One-way ANOVA revealed statistically significant differences among the experimental groups about the expression of GPR109 (F (3, 4) = 15.27, *p* = 0.0118), BDNF (F (3, 12) = 11.14, *p* = 0.0009), p-CREB (F (3, 8) = 18.05, *p* = 0.0006), SERT (F (3, 8) = 13.53, *p* = 0.0017) and PSD-95 (F (3, 12) = 6.971, *p* = 0.0057) (Fig. 16 B-F). Western blotting analysis showed that systemic administration of rotenone promoted reduced GPR109 levels in the prefrontal cortex (PD vs. CONTROL, *p* = 0.0210, Fig. 16A and B). Treatment with fluoxetine significantly increased the GP109 levels (PD + FLU vs. PD, *p* = 0.0218, Fig. 16A and B).

The PD group showed a decrease of phosphorylated CREB levels (cyclic-AMP response element binding protein) (PD vs. CONTROL, *p* = 0.0168, Fig. 16A and C), BDNF (PD vs. CONTROL, *p* = 0.0014, Fig. 16A and D), serotonin transporter (SERT) (PD vs. CONTROL, *p* = 0.0023, Fig. 16A and E), and PSD-95 (PD vs. CONTROL, *p* = 0.0169, Fig. 16A and F). In contrast, prebiotics increased the levels of p-CREB (PD + PREB vs. PD, *p* = 0.0012, Fig. 16A and C), BDNF (PD + PREB vs. PD, *p* = 0.0039, Fig. 16A and D), SERT (PD + PREB vs. PD, *p* = 0.0282, Fig. 16A and E), and PSD-95 (PD + PREB vs. PD, *p* = 0.0073, Fig. 16A and F) in the prefrontal cortex, indicating higher serotonin levels and neuroplasticity. Fluoxetine also increased p-CREB (PD + FLU vs. PD, *p* = 0.0008, Fig. 16A and C), SERT (PD + FLU vs. PD, *p* = 0.0028, Fig. 16A and E), and PSD-95 (PD + FLU vs. PD, *p* = 0.0228, Fig. 16A and F).

## Discussion

The rotenone-induced Parkinson’s disease model, which is sub-acute and rapidly progressive, with high variability and a limited therapeutic window, making delayed interventions challenging (Betarbet et al. 2000; Cannon et al. 2009; Johnson and Bobrovskaya 2015). Accordingly, many studies using this model adopt early or concomitant intervention strategies (Zhao et al. 2017; Siracusa et al. 2020), with gut dysbiosis and inflammatory signaling occur early in rotenone-induced pathology, supporting interventions during model induction (Dodiya et al. 2020). In the present study, we treated with a combination of prebiotics since previous studies corroborate the complementary and potentially synergistic effects of combining FOS + GOS, rather than each in isolation, in modulating the gut microbiota, short-chain fatty acid production, and behavioral outcomes, particularly in physiological or pathological conditions (Burokas et al. 2017).

The rotarod and open field tests are widely used to validate experimental PD models, in which bradykinesia and postural instability or loss of balance are pathognomonic features (Arab et al. 2021; Mendonça et al. 2022a,b). When treated with prebiotics simultaneously with rotenone, animals did not develop motor deficits, indicating a preventive effect of FOS + GOS on both parameters. To date, there are no studies directly evaluating prebiotics such as FOS and GOS in experimental model of DP. However, we can compare our results with studies that applied probiotics as therapy. Probiotics are strains whose function includes the fermentation of prebiotic fibers as an energy source, and some studies have shown that probiotics can attenuate or even prevent the motor symptoms of PD (Hsieh et al. 2020; Sun et al. 2021; Sancandi et al. 2023; Parra et al. 2023).

Αlpha diversity can be used to calculate species composition within samples, including two-dimensional information on number and abundance, while beta diversity can be used to study species composition among communities (Parikh et al. 2020). In the present study, alpha and beta diversity did not change significantly between groups, although there was a trend for α diversity to decrease in the PD-induced group and a tendency to return to baseline levels in the prebiotic-treated group. Despite this, rotenone promoted intestinal dysbiosis by altering groups of bacteria that play roles in intestinal homeostasis, while treatment with prebiotics promoted an increase in beneficial bacterial groups.

According to previous observations, the increased abundance of the *Firmicutes* phylum is associated with PD, whereas the abundance of the *Bacteroidetes* phylum is reduced, characterizing a higher *Firmicutes/Bacteroidetes* ratio (Yang et al. 2018). The present results confirmed the increased abundance of the *Firmicutes* phylum in the PD group, which was reversed after treatment with prebiotics and fluoxetine. Our results also showed that PD group had reduced the relative abundance of the *Bacteroidetes* phylum compared to the CONTROL group, confirming previous data (Li et al. 2019a). Accordingly, Unger et al. 2016a stated that the reduction of abundance of *Bacteroidetes* is accompanied by a reduction in SCFAs concentration in PD patients (Unger et al. 2016b). In this study, we demonstrate that FOS and GOS trends increase the relative abundance of Bacteroidetes compared to the PD group.

The *Actinobacteria* phylum includes some bacterial species related to the production of substrates for butyrate synthesis (Rivière et al. 2016). Interestingly, the present results showed that PD group also showed a reduction in the *Actinobacteria* phylum, whereas the prebiotics reversed this taxon alteration. Moreover, a higher abundance of the genus *Lactobacillus* observed in the PD group agrees with data obtained in a meta-analysis of the intestinal microbiome of Parkinson’s disease, in which the genus *Lactobacillus* and the family *Lactobacillaceae* were the most strongly enriched taxa in PD in the six clinical studies that reanalyzed (Romano et al. 2021). The intestinal abundance of *Lactobacillus* and other genera, including *Bifidobacterium*, also have been positively correlated with Crohn’s disease patients (Wang et al. 2014; Lewis et al. 2015). *Lactobacillus* strains are low abundant members of the gut human microbiota (5 to 0.1% of the proximal and distal gut, respectively), and their abundance is correlated positively or negatively with human diseases and chronic conditions (Heeney et al. 2018). Here, we show that treatment with FOS and GOs helped to maintain intestinal homeostasis by modulating the relative abundance of the *Lactobacillus* genus.

In contrast, a low abundance of the *Bacteroides* genus has been described in PD patients (Li et al. 2019b). As gut commensals, *Bacteroides* spp. have multiple roles; they can protect from pathogens by mucins production and metabolize polysaccharides and oligosaccharides, providing acetate and other substrates, such as lactate, for butyrate-producing bacteria (Louis and Flint 2017). The PD group showed reduced abundance of the *Bacteroides* spp, which was restored by treatment with FOS and GOS.

Treatment with prebiotics also promoted higher levels of *Alistipes ssp* and *Lactobacillus reuteri* compared to the PD group*,* which are known to produce acetate, propionate and butyrate (Parker et al. 2020; Cheng et al. 2022; Frolova et al. 2022). A decrease in the relative abundance of these bacteria has been reported in PD models (Stewart et al. 2009; Van Kessel and El Aidy 2019; Hong et al. 2021; Huang et al. 2021).

*L. reuteri* has a potent ability to produce tryptophan metabolites that activate host aryl hydrocarbon receptors (AHR) in epithelial tissue, which has been found to be essential for promoting the intestinal epithelial barrier and control of opportunistic pathogens (Zelante et al. 2013). Other authors also showed that *L. reuteri* promoted improvement in motor function, reduced oxidative stress, and attenuated the loss of dopaminergic neurons in the substantia nigra and striatum in a 6-OHDA-induced PD model (Nápoles-Medina et al. 2023). Moreover, these lactobacilli could be associated with producing SCFAs in the gut, particularly butyrate, through the fermentation of dietary fiber such as FOS and GOS. Thus, the increased abundance of the *L. reuteri* genus observed in the PD + Prebiotic group can be associated with improving PD symptoms and increasing serum and brain butyrate levels.

*Helicobacter* species are frequently used to model microbial triggers of colonic inflammation associated with developing inflammatory bowel disease (IBD) and neoplasia (Chichlowski et al. 2008). Moreover, *H. hepaticus* induces α-synuclein pathology, gut and brain inflammation, triggering dopaminergic degeneration and motor disorders in mice with Parkinson’s disease (Ahn et al. 2022). The results showed that FOS and GOS also significantly reduced the abundance of the *Helicobacter* genus and *Helicobater hepaticus*, positively correlating with improving inflammatory and motor dysfunctions in the PD-mice model.

SCFAs are metabolites responsible for maintaining intestinal homeostasis by modulating the integrity of the epithelial barrier through immune regulation. They are energy sources for colonocytes by activating G-protein-coupled receptors (GPRs) (Mohamed Elfadil et al. 2023). Activation of GPR43 promotes intestinal barrier integrity through mucus production and the expression of tight junction proteins (such as claudins and occludins), in addition to inhibiting the production of pro-inflammatory cytokines and promoting the differentiation of regulatory T cells (Tregs) (Maslowski et al. 2009; Sina et al. 2009). Our results showed that treatment with FOS and GOS strengthened the epithelial barrier, regulating the expression of occludin and zonula occludens, as well as reducing inflammatory cytokines through GPR3 signaling.

Butyrate serves as an agonist for GPR41, GPR43, and GPR109 (Siddiqui and Cresci 2021), and among SCFAs, it has received particular attention for its important anti-inflammatory and neuroprotective effects (Dang et al. 2021a; Mohamed Elfadil et al. 2023; Paiva et al. 2017; Getachew et al. 2020). In addition, butyrate also promotes the expression of anti-inflammatory factors through GPR43 signaling (Kibbie et al. 2021a; Dang et al. 2021b). Moreover, butyrate can enter cells through the solute carrier family 5, member 8 (SLC5A8) transporters, acting as a histone deacetylase (HDAC) inhibitor, which inhibits NFkB signaling and thereby inhibits inflammatory cytokine production. According to in vitro studies, butyrate significantly decreased intestinal lamina propria CD4 T cell activation and inflammatory cytokine compared to acetate and propionate through histone deacetylase (HDAC) inhibition and GPR43 signaling(Kibbie et al. 2021b).

Intestinal dysbiosis plays a relevant role in promoting intestinal and systemic inflammation, promoting synucleinopathies. Bacterial endotoxins, such as lipopolysaccharide (LPS), induce gut and systemic inflammation that alters the conformation of α-synuclein, promoting the formation of distinct pathological fibrils. These fibrils have a greater capacity for propagation and aggregation, suggesting that inflammation induced by microbial agents may accelerate the deposition of aggregates in synucleinopathies (Kim et al. 2016; Sampson et al. 2016). Beyond dysbiosis, rotenone promoted accumulation of intestinal phospho-α-synuclein and inflammation in colonic tissue with activation of NFkB and production of IL1-β. Inflammatory markers such as IL1-β associated with phospho-α-synuclein inclusions have also been found in intestinal biopsies of patients with PD (Devos et al. 2013). Another study also showed that a PD model induced by rotenone (30 mg/kg/day) for 7 days also promoted intestinal inflammation through the production of IL1-β and p-NFkB associated with dysbiosis, which probably contributes to the milieu intestinal inflammation (Zhao et al. 2021a, 2021b). Our results demonstrated that treatment with FOS and GOS, besides attenuating phospho-α-synuclein aggregation, also reduced the expression of NFkB and IL1-β, thus decreasing gut inflammation and maintaining gut immunity.

The SCFAs have also been reported in several studies to benefit the central nervous system (Lan et al. 2024), especially for their anti-inflammatory effects and maintenance of blood–brain barrier integrity (Chen et al. 2017; Chakraborty et al. 2024). Signaling through GPR109A (a butyrate receptor) attenuates LPS-induced microglial reactivity and NFkB levels, which in turn reduces the expression of several inflammatory mediators, such as iNOS, COX-2, TNF-α, IL-1β, and IL-6 (Fu et al. 2015; Giri et al. 2019). Accordingly, butyrate reduces hippocampal microgliosis and improves depressive-like behaviors secondary to neuroinflammation (Yamawaki et al. 2018). Several studies demonstrated that butyrate plays a role in neurodegenerative diseases including types of dementia such as Alzheimer’s disease(Skonieczna-żydecka et al. 2018; Marizzoni et al. 2020; Stadlbauer et al. 2020), and Parkison’s disease (Qiao et al. 2020; Liu et al. 2024).

Interestingly, PD patients present lower concentrations of butyrate in feces compared to healthy controls (Liu et al. 2024), and similar results were reported from ALS patients (Rowin et al. 2017), and mouse models of AD (Tran et al. 2019). Other studies showed that lower fecal butyrate is linked to epigenetic changes and depressive symptoms in PD patients (Xie et al. 2022). Low butyrate levels in PD patients are associated with increased “leak gut” and gut inflammation (Xie et al. 2022). An open-label, non-randomized PD patients study showed that a diet supplemented with inulin, a fructan-type saccharide such as FOS, resulted in beneficial biological changes in the microbiota, SCFAs, inflammation, and neurofilament light chain (Hall et al. 2023). The present results showed that FOS and GOS promoted a significant increase in serum and brain butyrate levels, as well as GPR109 brain expression, which may have attenuated neuroinflammation and depressive-like behavior.

We also found that rotenone decreased the expression of tyrosine hydroxylase (TH), the enzyme involved in dopamine synthesis, and increased the phosphorylation of α-synuclein both in the substantia nigra and colon. The decrease in TH reflects the death of SNpc dopaminergic neurons, a PD neuropathological marker, as it correlates to α-synuclein aggregation. The accumulation of α-synuclein forms cytoplasmic clusters called Lewy bodies within the remaining SNpc dopaminergic neurons (Colla et al. 2012; Choi et al. 2013). In addition, phospho-α-synuclein accumulation was also demonstrated in the gut of PD animals, which could reach the brain through gut-brain axis communication (Kelly et al. 2014; Seo et al. 2023). The precise role of toxic forms of α-synuclein in the etiology of PD is unclear. However, data generated from in vivo and in vitro models show that aggregated forms of α-synuclein may contribute to the neurodegenerative process in PD by interfering with lysosomal and mitochondrial function, impairing autophagy, vesicular homeostasis, and microtubule transport. Our results confirm those obtained in experimental PD models induced by rotenone in vitro (Shin and Chung 2020) and in vivo (Dodiya et al. 2020). Furthermore, for the first time is demonstrated that prebiotics and fluoxetine can reduce α-synuclein accumulation in the substantia nigra and colon of rotenone-induced animals.

Previous data from our group have already demonstrated that substantia nigra is affected by the neuroinflammation in this PD model (Mendonça et al. 2022a), and this data is also corroborated in other animal models (Sampson et al. 2016; Ma et al. 2023; Geng et al. 2023). Microglia are resident cells of the central nervous system, mainly responsible for the production of inflammatory mediators, and this production begins from the activation of the inflammatory phenotype of microglia (Gao et al. 2023). Microglial reactivity (microgliosis) is assessed by several markers, including IBA1 (Kustrimovic et al. 2018). In the present study, we found an increase in IBA1 expression in animals induced with rotenone (microgliosis) and a reversal of this increase in diseased animals treated with prebiotics. In our study, prebiotics also reduced rotenone-induced astrocyte immunoreactivity. Astrocytic reactivity, revealed by increased optical density for GFAP, is also part of a neuroinflammatory response. A recent study by Yin et al. 2023 studying an A30P-mutant human α-synuclein transgenic PD mice demonstrated that G-protein signaling 5 (RGS5) promotes neurodegenerative process through augmenting tumor necrosis factor receptor (TNFR) signaling in astrocytes (Yin et al. 2023). Interestingly, it is well-known that butyrate inhibits the activation of NFκB via HDAC inhibition or GPR109 signaling, which down-regulates the expression of pro-inflammatory cytokines (Fu et al. 2015; Giri et al. 2019), and promotes the expression of anti-inflammatory factors (Dang et al. 2021a). Therefore, prebiotic treatment reduced CNS neuroinflammation and synuclein aggregation, possibly through SCFAs effects, particularly butyrate. These results align with previous studies using other alternative treatments in PD models (Cai et al. 2022; Mendonça et al. 2022b; Zamanian et al. 2022).

The neuroinflammatory component in depression contributes to the pathological process of depression. Neuroinflammatory mediators are also observed in PD models with depressive-like behavior and contribute to the neurodegeneration characteristic of PD (Kamińska et al. 2017; Okano et al. 2019; Zhao et al. 2021a). In the analysis of depressive-like behavior, our data showed that animals with PD presented lower sucrose preference (SPT) and longer immobility time. Some studies have already shown the applicability of paradigms used in experimental depression research. In the sucrose preference test (SPT), anhedonia in the animal is measured by the reduction in sucrose consumption compared to basal levels; anhedonic rodents show decreased preference for the sucrose solution, probably due to a reduced sense of reward from the sweet taste, in the same way, the antidepressant efficacy is evaluated by the greater preference for sucrose solution (Wang et al. 2009; Schintu et al. 2012; Mendonça et al. 2022b, a). In the tail suspension test (TST), an endophenotype called “despair-like behavior” is evaluated, which is proportional to the time of immobility (Schintu et al. 2012). Our results demonstrated that the rotenone-induced PD model develops anhedonic and despair-like behavior or depressive-like behavior, confirming recent studies using 6-OHDA-induced PD models (Bonato et al. 2018; Vecchia et al. 2021). Our data also showed, for the first time, that prebiotics FOS and GOS were able to inhibit the development of anhedonic or despair-like depressive behavior in PD rotenone-induced mice. Interestingly, prebiotics showed similar results to the fluoxetine used here as the golden standard for depression treatment. In addition, prebiotics also reduced iNOS, NFkB, and the inflammation mediator IL-1β and increased brain serotonin and serotonin transporter (SERT) levels, which the reduced levels are associated with depression (Borroto-Escuela et al. 2021).

In the present study, treatment with FOS and GOS increased cAMP response element binding protein (CREB), brain-derived neurotrophic factor (BDNF), and postsynaptic density protein 95 (PSD-95) expression in the prefrontal cortex, which were depleted by rotenone treatment. The role of serotonin in depression and the loss of neuroplasticity in the prefrontal cortex are related to depression (Borroto-Escuela et al. 2021). Previous studies have shown that increased neuroplasticity through phospho-CREB and high serotonin levels resulted in improvement in spatial restraint stress (SRS)-induced depression in mice (Duman et al. 2016; Kraus et al. 2017; Li et al. 2019c). PSD-95 is a key protein in the synapse that plays a vital role in the PSD organization, regulation of receptor trafficking, synaptic plasticity, neural development, and cognitive function. Furthermore, PSD-95 is associated with the improvement of depressive-like behavior in a model of depression induced by maternal separation model in rats (Deng et al. 2022). Taken together, FOS and GOS treatment prevented neurodegeneration and improved neuroplasticity associated with depression comorbid with Parkinson’s disease, possibly through SCFAs effects, particularly butyrate.

Thus, FOS and GOS may be a novel nutritional therapeutic approach for future interventions in the treatment of depression associated with Parkinson’s disease. Clinical studies with these two prebiotics should be performed to achieve human translational effects.

However, there are some limitations of the present study including the use of only male animals, the absence of quantification of other short fatty acids (SCFAs), the concomitant administration of prebiotics with model induction, and the combined administration of FOS and GOS instead of testing each prebiotic individually. However, future studies should address sex differences, treatment paradigms, and the individual contributions of each prebiotic.

## Conclusion

**Fig. 17:**
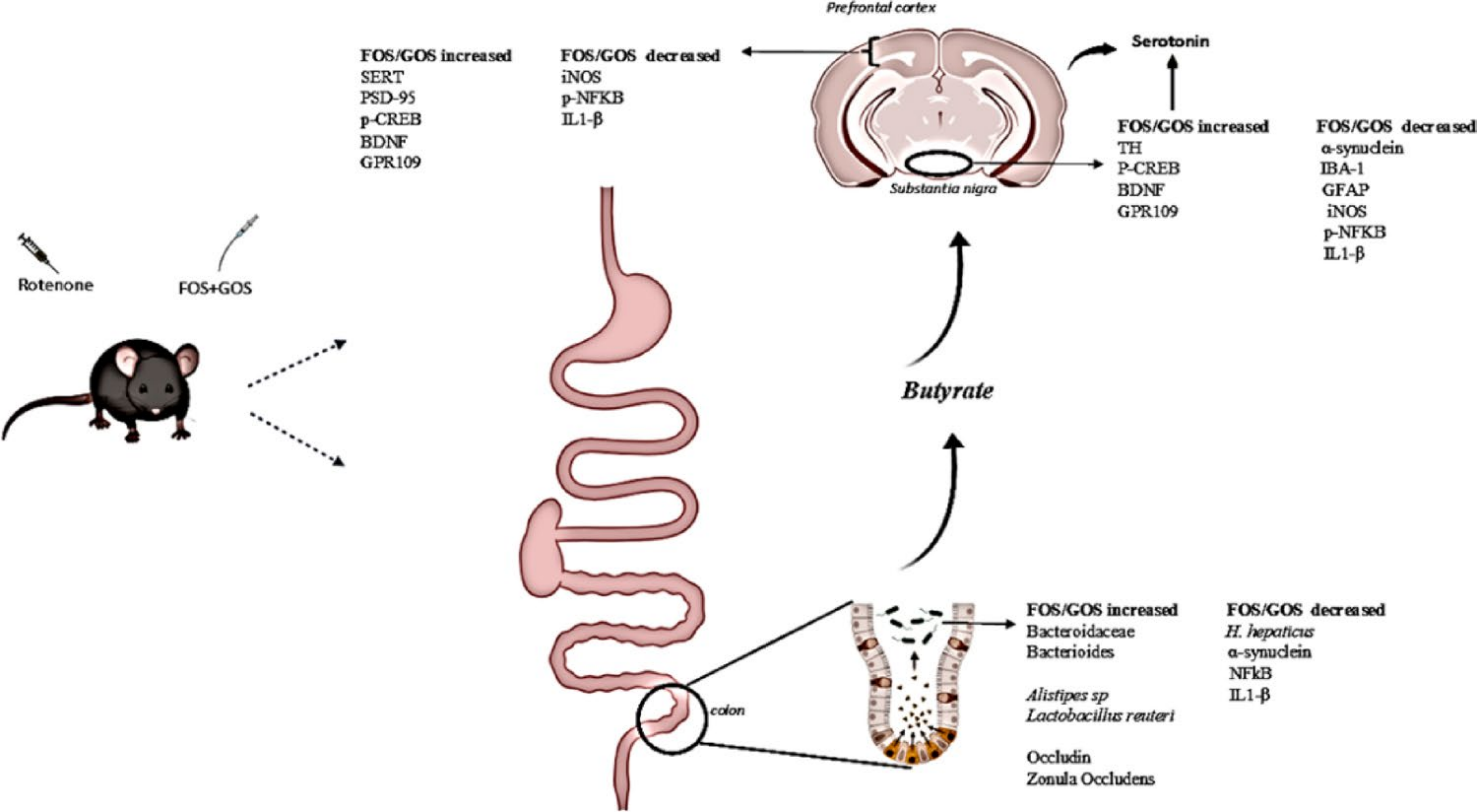
FOS and GOS act in the intestine by increasing the relative abundance of short-chain fatty acid-producing bacteria Bacteroidaceae, Bacteroides, *Alistipes *sp, *Lactobacillus reuteri* and reducing the pro-inflammatory species such as *H. hepaticus*. The prebiotics also decreased α-synuclein, p-NFKB, and IL1-β, increasing occludin and GPR43 levels, thus reducing gut inflammation and permeability. In the Substantia nigra, FOS and GOS increased the expression of TH and brain neuroplasticity factors p-CREB and BDNF, in addition to reducing IBA-1, GFAP, p-NFKB, IL1-B, iNOS and α-synuclein. In the prefrontal cortex, FOS and GOS also reduced the expression of inflammatory markers p-NFKB, IL1-β, and iNOS and promoted increased neuroplasticity by expressing SERT, PSD-95, BDNF, and p-CREB. Finally, FOS and GOS also increased brain serotonin levels

This study was the first to test a potential nutraceutical strategy of FOS and GOS in a model of Parkinson’s disease, possibly mediated through SCFAs effects. The results showed that this combination improved motor performance and depressive-like behavior by improving brain serotonin levels and its respective receptor (SERT). Treatment with FOS and GOS increased butyrate-producing gut bacteria and higher levels of serum and brain butyrate and GPR109 brain levels. Moreover, prebiotics reduced gut inflammation via NFkB inhibition and decreased α-synuclein deposition, whereas they increased zonula occludens and occludin, reducing gut permeability. FOS and GOS also reduced neuroinflammation in the prefrontal cortex and substantia nigra and promoted neuroplasticity through increased expression of p-CREB, BDNF, and postsynaptic protein PSD-95. Thus, FOS and GOS positively modulated the microbiota-brain axis and, therefore, may be used in the future as adjuvant nutraceutical strategies for the treatment of Parkinson’s disease. Figure 17 summarizes the effects of FOS and GOS reported in this study.

## Data Availability

No datasets were generated or analysed during the current study.
